# Genome-wide screen and multi-omics analysis reveal *OGT1* participate in the biosynthesis of safflower flavonoid glycosides

**DOI:** 10.1093/hr/uhae261

**Published:** 2024-09-16

**Authors:** Bin Xian, Yanxun Zhou, Yueying Hu, Yanni Peng, Xiaominting Song, Ziqing Xi, Yuhang Li, Jie Yan, Chaoxiang Ren, Jin Pei, Jiang Chen

**Affiliations:** State Key Laboratory of Southwestern Chinese Medicine Resources, Chengdu University of Traditional Chinese Medicine, 1166 Liutai Avenue, Wenjiang District, Chengdu 611137, Sichuan, China; College of Pharmacy, Chengdu University of Traditional Chinese Medicine, 1166 Liutai Avenue, Wenjiang District, Chengdu 611137, Sichuan, China; State Key Laboratory of Southwestern Chinese Medicine Resources, Chengdu University of Traditional Chinese Medicine, 1166 Liutai Avenue, Wenjiang District, Chengdu 611137, Sichuan, China; College of Pharmacy, Chengdu University of Traditional Chinese Medicine, 1166 Liutai Avenue, Wenjiang District, Chengdu 611137, Sichuan, China; State Key Laboratory of Southwestern Chinese Medicine Resources, Chengdu University of Traditional Chinese Medicine, 1166 Liutai Avenue, Wenjiang District, Chengdu 611137, Sichuan, China; College of Pharmacy, Chengdu University of Traditional Chinese Medicine, 1166 Liutai Avenue, Wenjiang District, Chengdu 611137, Sichuan, China; State Key Laboratory of Southwestern Chinese Medicine Resources, Chengdu University of Traditional Chinese Medicine, 1166 Liutai Avenue, Wenjiang District, Chengdu 611137, Sichuan, China; College of Pharmacy, Chengdu University of Traditional Chinese Medicine, 1166 Liutai Avenue, Wenjiang District, Chengdu 611137, Sichuan, China; State Key Laboratory of Southwestern Chinese Medicine Resources, Chengdu University of Traditional Chinese Medicine, 1166 Liutai Avenue, Wenjiang District, Chengdu 611137, Sichuan, China; College of Pharmacy, Chengdu University of Traditional Chinese Medicine, 1166 Liutai Avenue, Wenjiang District, Chengdu 611137, Sichuan, China; State Key Laboratory of Southwestern Chinese Medicine Resources, Chengdu University of Traditional Chinese Medicine, 1166 Liutai Avenue, Wenjiang District, Chengdu 611137, Sichuan, China; College of Pharmacy, Chengdu University of Traditional Chinese Medicine, 1166 Liutai Avenue, Wenjiang District, Chengdu 611137, Sichuan, China; State Key Laboratory of Southwestern Chinese Medicine Resources, Chengdu University of Traditional Chinese Medicine, 1166 Liutai Avenue, Wenjiang District, Chengdu 611137, Sichuan, China; College of Pharmacy, Chengdu University of Traditional Chinese Medicine, 1166 Liutai Avenue, Wenjiang District, Chengdu 611137, Sichuan, China; State Key Laboratory of Southwestern Chinese Medicine Resources, Chengdu University of Traditional Chinese Medicine, 1166 Liutai Avenue, Wenjiang District, Chengdu 611137, Sichuan, China; College of Pharmacy, Chengdu University of Traditional Chinese Medicine, 1166 Liutai Avenue, Wenjiang District, Chengdu 611137, Sichuan, China; The State Bank of Chinese Drug Germplasm Resources, Chengdu University of Traditional Chinese Medicine, 1166 Liutai Avenue, Wenjiang District, Chengdu 611137, China; State Key Laboratory of Southwestern Chinese Medicine Resources, Chengdu University of Traditional Chinese Medicine, 1166 Liutai Avenue, Wenjiang District, Chengdu 611137, Sichuan, China; College of Pharmacy, Chengdu University of Traditional Chinese Medicine, 1166 Liutai Avenue, Wenjiang District, Chengdu 611137, Sichuan, China; The State Bank of Chinese Drug Germplasm Resources, Chengdu University of Traditional Chinese Medicine, 1166 Liutai Avenue, Wenjiang District, Chengdu 611137, China; State Key Laboratory of Southwestern Chinese Medicine Resources, Chengdu University of Traditional Chinese Medicine, 1166 Liutai Avenue, Wenjiang District, Chengdu 611137, Sichuan, China; College of Pharmacy, Chengdu University of Traditional Chinese Medicine, 1166 Liutai Avenue, Wenjiang District, Chengdu 611137, Sichuan, China; The State Bank of Chinese Drug Germplasm Resources, Chengdu University of Traditional Chinese Medicine, 1166 Liutai Avenue, Wenjiang District, Chengdu 611137, China; State Key Laboratory of Southwestern Chinese Medicine Resources, Chengdu University of Traditional Chinese Medicine, 1166 Liutai Avenue, Wenjiang District, Chengdu 611137, Sichuan, China; College of Pharmacy, Chengdu University of Traditional Chinese Medicine, 1166 Liutai Avenue, Wenjiang District, Chengdu 611137, Sichuan, China; The State Bank of Chinese Drug Germplasm Resources, Chengdu University of Traditional Chinese Medicine, 1166 Liutai Avenue, Wenjiang District, Chengdu 611137, China

## Abstract

Safflower, an economic crop, is renowned for its flowers, which are widely used in medicines for treating cardiovascular and cerebrovascular diseases and in dyes for food and industry. The utility of safflower depends on its flavonoid glycosides. Therefore, the biosynthesis of safflower flavonoid glycosides has been a focus of attention, but the present mechanisms remain poorly understood. This study aims to identify functional genes associated with flavonoid glycoside biosynthesis in safflower through a comprehensive approach that integrates whole-genome screen and multi-omics correlation studies. CYP and UGT are two crucial genes families involved in flavonoid glycoside biosynthesis. We have screened 264 CYP genes and 140 UGT genes in the genome of safflower and conducted analyzes including phylogenetic relationships, conserved motifs, gene structures, *cis*-acting elements, and chromosome mapping, which provided extensive and comprehensive data on the CYP and UGT gene families. Integration of phenotype and metabolic data from safflower different tissues helped narrow down the screening by confirming that HSYA is synthesized only in flowers. Based on the gene expression patterns and phylogenetic analysis, *CtOGT1* was ultimately identified, which could catalyze the generation of glycosides using various flavonoid substrates and exhibited strong substrate affinity. Moreover, molecular docking studies elucidated CtOGT1’s highly active intrinsic mechanism. In conclusion, this study effectively identified genes responsible for flavonoid glycoside biosynthesis in safflower through the integration of whole-genome screen and multi-omics analysis, established a comprehensive foundation of data, methodology, and experimental evidence for further elucidating the pathways of safflower flavonoid glycoside biosynthesis.

## Introduction

Safflower (*C. tinctorius* L.), a member within the Asteraceae family, with a cultivation history of over 4500 years and holds significant value as an economic crop. It is distributed in multiple countries including the USA, China, Mexico, Japan, India, and Egypt [1]. Safflower flowers contain a wide range of flavonoid glycosides. Studies have indicated that these compounds provide notable therapeutic advantages for treating cardiovascular and cerebrovascular disorders. For example, hydroxysafflor yellow A (HSYA) can alleviate issues such as cerebral ischemia–reperfusion injury, thrombosis, and arteriosclerosis. Safflower is utilized as a medicinal plant in numerous countries and regions. In China, it is a primary ingredient in over 300 traditional formulations, including Danhong injection, which is administered to treat stroke, coronary heart disease, and angina [2, 3]. In Japan, safflower is essential in Kampo preparations, which are employed to improve blood circulation and reduce blood stasis. Additionally, flavonoid extracts from safflower are used as healthful natural pigments. In ancient Egypt, Japan, and India, safflower-derived flavonoids were crucial for coloring traditional garments. These pigments are also employed in natural food colorings for products like bread, cakes, biscuits, candies, and beverages [4]. Industrially, they are utilized in the coloring of pharmaceuticals and cosmetics [5].

Flavonoid glycosides in safflower can be classified into two main categories: one includes the flavonoids and flavonols commonly found in most plants, while the other comprises chalcones and their glycosides, including HSYA, hydroxysafflor yellow B, and saffloquinoside A, which are also known as pigmented components, unique to safflower [6]. The biosynthetic pathways of flavonoids and flavonols are similar to those of model plants and crops and their biosynthetic pathway has been elucidated. There are many reports on the shared genes in the biosynthesis pathways of these flavonoid compounds, such as *CHS* [7], *CHI* [8], *F3H* [9], and *F3’5’H* [10]. However, the biosynthesis pathway of these unique components, especially flavonoid glycosides, in safflower is still unclear. Based on the chemical structures of these compounds, their biosynthesis is believed to proceed through glycosylation and hydroxylation on the basis of chalcones (Fig. S1). In recent years, our work has centred around the investigation of biosynthesis pathways for these components, such as Ren *et al*. [11] screened the glycosyltransferase gene *UGT3*, and Chen *et al*. [12] screened *CtCGT1* in safflower. At the same time, many researchers were also devoted to elucidating the biosynthetic pathways flavonoid glycosides in safflower. For example, Xu *et al*. [13] cloned and confirmed the roles of glycosyltransferase (GT) genes in glycoside biosynthesis, particularly the bifunctional GT. Wang *et al*. [14] identified and validated a new cytochrome oxidase gene in safflower. However, current gene screening predominantly references genes reported in other species and lacks a comprehensive whole-genome perspective specific to safflower. This approach provides limited information for elucidating the biosynthetic pathway of flavonoid glycosides in safflower, leaving the pathway unclear.

Currently, the discovery of functional genes largely utilizes methods like differential gene screening through RNA-seq coupled with metabolomics, as well as strategies such as homology comparison and mining of gene clusters based on genomic data [15]. In plant natural product biosynthesis, increased gene expression levels are frequently positively correlated with the accumulation of bioactive substances. Transcriptomics and metabolomics track these changes in gene expression and substance accumulation across various tissues, developmental stages, and environmental stress. Integrating transcriptomics and metabolomics is a vital approach for investigating the regulatory networks and physiological mechanisms underlying plant development [16] and is widely employed in the study of numerous economical crops. For example, Li *et al*. [17] integrated spatiotemporal metabolomic and transcriptomic data to construct a metabolic regulation network of tomatoes at different tissues and growth stages; Wu *et al*. [18] conducted a joint transcriptomic and metabolomic analysis of immature and mature blackberry fruits to decipher the flavonoid biosynthesis mechanism. However, this approach often halts at the screening of differentially expressed genes or differential metabolites and remains theoretical in terms of elucidating specific molecular mechanisms. Gene screening based on genomic data typically aims to elucidate specific gene functions [19]. However, homology comparison used to screen functional genes has many limitations, often necessitating functionally genes from closely related species or plants within the same family or genus as references. For example, Zhao *et al*. [20] screened functional genes in *Scutellaria baicalensis* Georgi by comparing genes from four other species within the Lamiaceae family, and Hagel *et al*. [21] also used genes from related plants within the same family to screen functional genes in *Macleaya cordata*. The whole genome contains extensive and comprehensive information on gene families, and the integration with multi-omics analyses can be used effectively and efficiently for genome-phenotype associations and gene identification [22]. This method has been proven effective in various economic crops such as wheat, rapeseed [23], and soybean [24].

This research aimed to pinpoint the essential genes responsible for the biosynthesis of flavonoid glycosides in safflower by cataloging all members of the CYP and UGT superfamilies present in the safflower genome and got the detailed information of their phylogeny, conserved motifs, gene structures, and promoter *cis*-acting elements. Phenotype–metabolite association analyses were conducted, based on the presence of a red substance which was suspected to be HSYA, in the roots, stems, leaves, and flowers of safflower. Transcriptomic analyses of safflower were performed under different developmental stages, tissues, light intensities, and methyl jasmonate (MeJA) treatments, which can influence the accumulation of flavonoid glycosides in safflower. By integrating whole-genome and multi-omics association analyses, candidate genes involved in the biosynthesis of safflower flavonoid glycosides were screened and their activity was validated. Overall, this study associates key gene families involved in the biosynthesis process of safflower flavonoid glycosides, phenotypic and metabolic characteristics of different tissues, and transcriptomic results under various conditions, providing a comprehensive strategy for the screening of functional genes in safflower flavonoid glycoside biosynthesis and offering methodological support for the elucidation of biosynthetic pathways of bioactive components in other plants.

## Results

### Identification of *CYPs* and *UGTs* in safflower genome

The safflower genome data were obtained from our previous study, and gene annotation was already performed [12]. We retrieved all relevant sequences based on Pfam annotation. CYPs are characterized by the Pfam domain PF00067 and UGTs are characterized by the Pfam domain PF00201. We identified a total of 264 transcripts belonging to CYP family and 140 transcripts associated with UGT family in safflower.

The protein lengths of CYP ranged from 303 to 612 amino acids, with molecular weights between 34.08 and 69.68 kDa, and theoretical isoelectric points from 5.42 to 10.15. Predictions for subcellular localization suggested that the majority of CYP proteins (260 out of 264) were situated within the endomembrane system. For UGTs, proteins exhibited a length variation, ranging from 303 to 612 amino acids, isoelectric points from 4.64 to 9.45, and molecular weights from 22.30 to 62.40 kDa. Subcellular localization showed that most UGT proteins were primarily found in the chloroplast (72 out of 140), with the second-largest group located at the cell membrane (19 out of 140). Comprehensive details and characteristics of CYPs and UGTs, including the instability index, aliphatic index, and the grand average of hydropathicity, are included in Tables S1 and S2.

### Phylogenetic analysis of CYPs and UGTs in safflower

**Figure 1:**
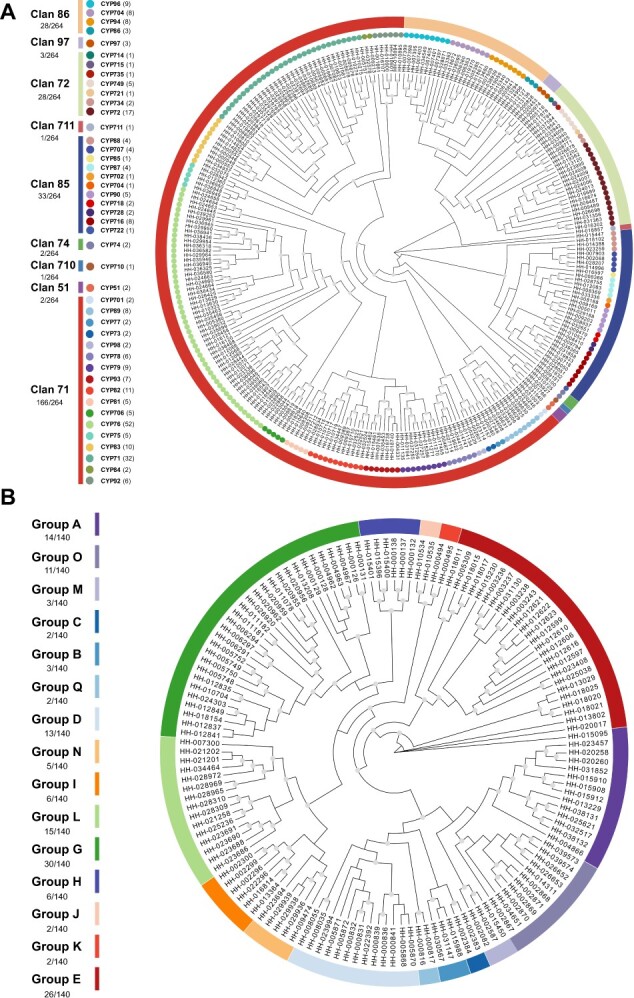
Phylogenetic analysis of CYPs and UGTs in safflower. To construct the maximum likelihood tree, protein sequences of CYPs and UGTs were utilized, with the analysis supported by 5000 bootstrap replicates. Bootstrap values greater than 0.7 are indicated by gray circles placed at the center of each branch. The clans and families of CYPs are distinguished by color strips and circles, respectively (**A**), while the groups of UGTs are marked using color strips (**B**).

To further explore the evolutionary relationships of CYPs and UGTs in safflower, an unrooted Maximum Likelihood (ML) tree was constructed based on a trimmed alignment, respectively. This analysis enabled the assignment of 264 CYP and 140 UGT protein sequences to specific clans or groups (Fig. 1, and details can be viewed in Table S3).

 In safflower, we identified nine CYP clans which encompass a total of 45 families. These clans were categorized into the A-type and non-A-type, according to their positions on the phylogenetic tree. The A-type consisted exclusively of clan 71, a multi-family clan that contains 17 families and a total of 166 genes. In contrast, the non-A-type included the other eight clans. Clans 51, 74, and 710 were grouped into one cluster, while clans 72, 86, 97, and 711 formed another cluster. Clan 85, however, stood alone as a single-clan cluster. Among the non-A-type clans, clans 72, 85, and 86 were multi-family clans. Clan 72 contained seven families with a total of 28 genes, clan 85 included eleven families with 33 genes, and clan 86 comprised four families with 28 genes; the other clans were single-family clans and each comprised 1 to 3 gene. Further details can be viewed in Fig. 1A.

 One hundred and forty UGT sequences of safflower were divided into 15 groups, group A, B, C, D, M, O, and Q were grouped into one cluster, group G, H, I, J, K, L, and N formed another cluster, group E constituted a single-group cluster. The largest group, designated as Group G, consisted of 30 gene members. In contrast, the smallest groups, labeled C, J, K, and Q, each contained only two members. The other groups had the following number of members: group A consisted of 14 members, group B had 3 members, group D contained 13 members, and group E comprised 26 members. Additionally, group H and Group I each had 6 members, Group L included 15 members, and both Group N had 5 members, and Group O had 11 members. The detail can be viewed in Fig. 1B.

### Conserved motifs, gene structures, and chromosomal mapping of CYPs and UGTs

**Figure 2:**
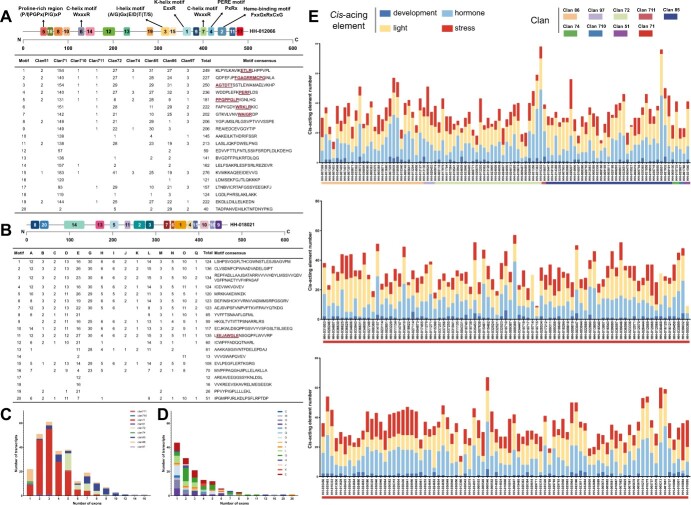
The allocation of conserved motifs and exons in nine clans of CYPs and fifteen groups of UGTs and the number of cis-acting elements in the promoter region of the nine clans of safflower CYPs. (A) Schematic diagram of conserved motifs in safflower CYP proteins, using HH-012066 as an example. And the distribution of 20 conserved motifs across nine CYP clans. The signature motifs, which contain functionally characterized domains, are indicated. N and C represent the N-terminal and C-terminal, respectively. Additionally, the exon distribution of transcripts in nine CYP clans is shown. (B) Schematic diagram conserved motifs in safflower UGT proteins, taking HH-018021 as an example, and the distribution of 20 conserved motifs in fifteen groups of UGTs. (C) Exon distribution of transcripts of CYP. (D) Exon distribution of transcripts of UG. (E) The predicted cis-acting elements were categorized into four groups: development, stress, hormone, and light responsiveness.

Using MEME software, we identified a total of 20 conserved motifs in both the CYP and UGT families of safflower. Overall, the distribution of these motifs varies significantly among different families or groups, while similar patterns are observed within the same family or group. In CYPs (Fig. 2A, and details can be viewed in Figs S2 and S3), a notable distinction was identified between A-type and non-A-type, with all 20 motifs present in A-type, while only motifs 6–19 appeared in non-A-type CYPs. Among these motifs, motifs 1, 2, 4, and 5 were highly conserved across all CYP clans. Specifically, motif 5 was predominantly located at the C-terminus, whereas motifs 1, 2, and 4 were primarily found at the N-terminus. And seven of them (motifs 1–7) contained the functionally characterized domains.

Motif 1 included the K-helix motif (ExxR). Motif 2 featured the heme-binding motif (FxxGxRxCxG). Motif 3 contained the I-helix motif ((A/G)Gx(E/D)T(T/S)). Motif 4 consisted of the PERE motif (PxRx). Motif 5 included the proline-rich region ((P/I)PGPx(P/G)xP). Motifs 6 and 7 comprised the C-helix motif (WxxxR). In UGTs (Fig. 2B, and details can be found in Fig. S4 and Fig. S5), motif preferences varied among groups. Only Group E contained all motifs, while other groups had only 12–16 motifs. Among the 20 motifs, motifs 1, 2, 3, 4, 5, 6, 10, and 11 were highly conserved and present in all UGT groups. Motifs 2, 3, and 5 were primarily located at the C-terminus, whereas motifs 1, 4, 6, 10, and 11 were mainly at the N-terminus. Motifs 17 and 18, being less conserved, appeared exclusively in Group E.

The structural distribution of exons, introns, coding sequences (CDS), and untranslated regions (UTRs) in CYPs and UGTs was also summarized to enhance understanding of the gene family’s structural evolution. The CDS-UTR constitution of non-A-type CYPs varied significantly compared to A-type CYPs, which exhibited similar distributions (Fig. 2C and Fig. S6). A-type CYPs contained 1–8 exons, with 59.6% (99 out of 166) having 2–3 exons. In contrast, in non-A-type CYP, the number of exons fluctuated widely. For example, family 97 exhibited 10 or more exons, family 85 ranged from 2 to 12 exons, and the remaining CYP families generally had fewer than 10 exons. In UGTs (Figs 2D and S7), a small number of genes (6 out of 140) had more than nine exons, while the majority (96 out of 140) contained between one and three exons. Specifically, Groups A (14 of 140), D (13 of 140), and J (2 of 140) had between one and six exons, whereas Groups B (3 of 140), C (2 of 140), K (2 of 140), L (15 of 140), and O (11 of 140) ranged from one to four exons.

Genetic localization on the chromosomes was mapped using the genome annotation data for safflower (Fig. S8). Out of 264 CYP genes, 157 genes, and out of 140 UGT genes, 111 genes were randomly distributed across 12 chromosomes. The residual genes could not be mapped to specific chromosomes and thus remained anchored on scaffolds [25]. The distribution of CYP and UGT genes on chromosomes exhibited uneven patterns. High densities of CYP and UGT genes were observed on chromosome 1, containing 56 and 47 genes, respectively. Conversely, the lowest distribution of CYP genes was found on chromosomes 5 and 7, each with only five genes. Meanwhile, the UGT genes showed minimal presence on chromosome 8, with only one gene.

### C*is*-acting elements analysis in the promoter region of CYPs and UGTs

To better analyze the transcriptional regulatory mechanisms of safflower’s CYP and UGT genes, a 2000 base pair (bp) sequence located upstream from the start codon (ATG) of each transcript was extracted for the prediction of cis-acting elements. In addition to the core elements, the cis-acting elements were categorized into four distinct groups: developmental elements, stress-related elements, hormonal elements, and light-responsive elements.

Out of the 32 predicted light-responsive elements, the four with the highest frequencies were Box 4 (90.9%, 240 out of 264), G-box (83.3%), GT1-motif (78.8%), and TCT-motif (58.7%), which confirmed that the light-responsive elements are the most abundant of the safflower CYP gene family promoters (Fig. 2E, additional details in Figs S9 and S10). This was followed by hormone-responsiveness *cis*-acting elements, the TGACG-motif and CGTCA-motif emerged as the most frequent, each occurring at a rate of 78.4%. This was closely followed by the abscisic acid-responsive *cis*-acting element (ABRE), which appeared in 78% of cases, and the ethylene-responsive element (ERE), occurring at a frequency of 59.5%. Among the *cis*-acting elements concerned with stress responses, ARE (90.9%), WRE3 (54.2%), LTR (48.9%), and TC-rich repeats (37.9%) were the most abundant. Specifically, ARE was essential for anaerobic induction, WRE3 served as a wound-responsive element, LTR was a low-temperature responsiveness element, and TC-rich repeats were linked to defence and stress responses. In safflower CYPs, several developmentally responsive *cis*-acting elements, including the CAT-box, circadian, HD-Zip 1, and GCN4_motif, were identified. These elements are associated with meristem expression, circadian control, differentiation of palisade mesophyll cells, and endosperm expression, respectively. They were found in 90 (34.1%), 58 (22%), 35 (13.3%), and 30 (11.4%) of CYPs.

The analysis of promoter sequences of UGTs, as illustrated in Figs S11, S12, and S13, revealed a significant diversity and abundance of light-responsive elements. A total of 33 light-responsive *cis*-acting elements were predicted, with the four most frequent being the G-box (83.6%), Box 4 (78.6%), GT1-motif (69.3%), and TCT-motif (57.9%). In safflower UGTs, the four most prevalent hormone-responsive *cis*-acting elements were ABRE, TGACG-motif, CGTCA-motif, and ERE. These *cis*-acting elements were associated with abscisic acid, MeJA, and ethylene responsiveness, respectively. Specifically, both the TGACG-motif and CGTCA-motif were linked to MeJA responsiveness. Their occurrence rates were as follows: ABRE at 82.9% (116 out of 140), TGACG-motif and CGTCA-motif each at 81.4% (114 out of 140), and ERE at 58.6% (82 out of 140). Stress-responsive *cis*-acting elements were also identified, including the most frequently occurring anaerobic-induction element (ARE, 93.6%), the wound-responsive element (WRE3, 57.9%), the low-temperature responsiveness element (LTR, 52.1%), and the drought-inducibility element (MBS, 40.7%). Several development-responsive *cis*-acting elements, including the AAGAA-motif, CAT-box, circadian, and GCN4_motif—were identified in UGTs, related to secondary xylem development, meristem expression, circadian control, and endosperm expression, respectively. These elements accounted for 62.1%, 30%, 19.3%, and 14.3% of the UGT numbers.

### Phenotypic and metabolic association analysis of flavonoids in different parts of safflower

**Figure 3:**
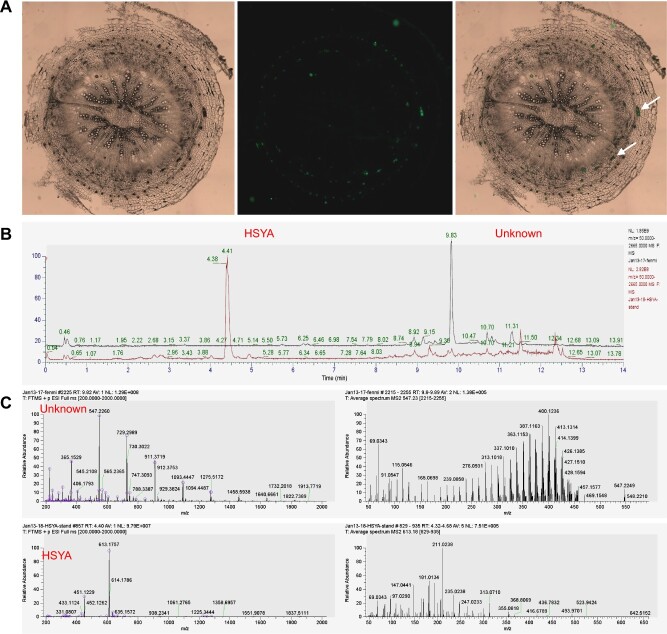
Identification of the RSs in safflower roots, stems and leaves. (A) The distribution of RS in safflower roots. From left to right are bright field, green fluorescent protein fluorescence and merged illumination, respectively. (B) The HPLC chromatogram of the RS and HSYA. (C) The mass spectra of the RS and HSYA.

After transverse sections of the safflower roots, stems, and leaves were made, a distinct red substance (RS) was observed to seep out. Then, the transverse sections of safflower roots were observed under a fluorescence microscope, revealing that the RS exhibited a distinct green fluorescence located between the endodermis and the cambium (Figs 3 and S19). The UPLC results revealed that the retention times of the RS and HSYA were completely different, and the MS results indicated a notable difference between the RS and HSYA in terms of molecular weights and mass spectra, indicating that the RS and HSYA were two completely different compounds. Furthermore, the metabolic analysis revealed that HSYA was absent from the flavonoid metabolism profiles of safflower roots, stems, and leaves, which further suggests that HSYA is a compound specifically biosynthesized in safflower flowers (Tables S5 and S6).

### Expression profiling of *CYP* and *UGT* genes of safflower under different development stages, light intensities, tissues, and MeJA treatment

Numerous factors influence gene expression. Promoter analysis indicates that development, light, hormones, and stress are the primary factors affecting the expression of CYP and UGT genes. In our preliminary studies, we found that developmental stages, light intensity, and MeJA treatment significantly influence the levels of two categories of flavonoid glycosides in safflower, moreover the types and contents of flavonoid glycosides vary considerably among different tissues. *Cis*-acting element analysis further confirmed the involvement of CYP and UGT in the biosynthesis of safflower flavonoid glycosides. Therefore, analyzing the expression patterns of CYP and UGT under these conditions will aid in identifying functional genes in future research.

Transcriptome data from safflower at various flowering stages, different light intensities, different tissues, and MeJA treatment were analyzed (Figs S14 and S15). *CYP* and *UGT* genes were separately grouped into clusters according to their expression patterns, with each cluster consisting of genes that showed similar expression profiles. The expression profiles of genes under different light intensities and at different developmental stages were categorized into six clusters. The expression profiling of genes under different light intensities and different developmental stages were classified into six clusters, and each cluster for CYP contained 15–69 genes and for UGT contained 13–32 genes (Figs S16A and S16B), respectively. Samples treated with MeJA, along with samples from various tissues, were analyzed and grouped into six clusters. Each CYP cluster contained 17–63 genes (Fig. S16C), while each UGT cluster included 11–31 genes (Fig. S17C).

In a transcriptomic cluster analysis of safflower flowers under different light intensity treatments, the expression of 127 *CYP* genes (from clusters 2, 4, and 5) and 60 *UGT* genes (from clusters 3, 4, and 6) showed an initial increase followed by a decrease or gradual rise; the *CYPs* including all members of clans 51 and 97, 64.29% of the members from clan 86, and 47.59% of the members from clan 71.; additionally, *UGTs* included more than 60% of the members from groups B, I, L, M, and O. In the cluster analysis of transcriptomic data from flowers at stages 1 to 4, we focused on genes that exhibited an overall increasing trend in expression levels; a total of 116 *CYP* genes were identified in clusters 2, 4, and 5, with members of clan 71 being the most abundant, accounting for 63.79%; 59 *UGT* genes were concentrated in clusters 2, 5, and 6, primarily from group E (18.64%), group G (27.12%), and group L (18.64%). Considering that flavonoid components in safflower are abundant in the flowers and could be induced by MeJA, we focused on the *CYP* genes in clusters 4, 5, and 6 (103 genes) and the UGT genes in clusters 3, 4, and 5 (65 genes); among the selected *CYP* genes, members of clan 71 were the most numerous, accounting for 57.28% of the total; among the selected *UGT* genes, apart from not containing any members from groups B, C, and Q, the proportion of members from other groups ranged from 3.08% to 21.54%.

### Discovery of uridine diphosphate glycosyltransferase in safflower

**Figure 4:**
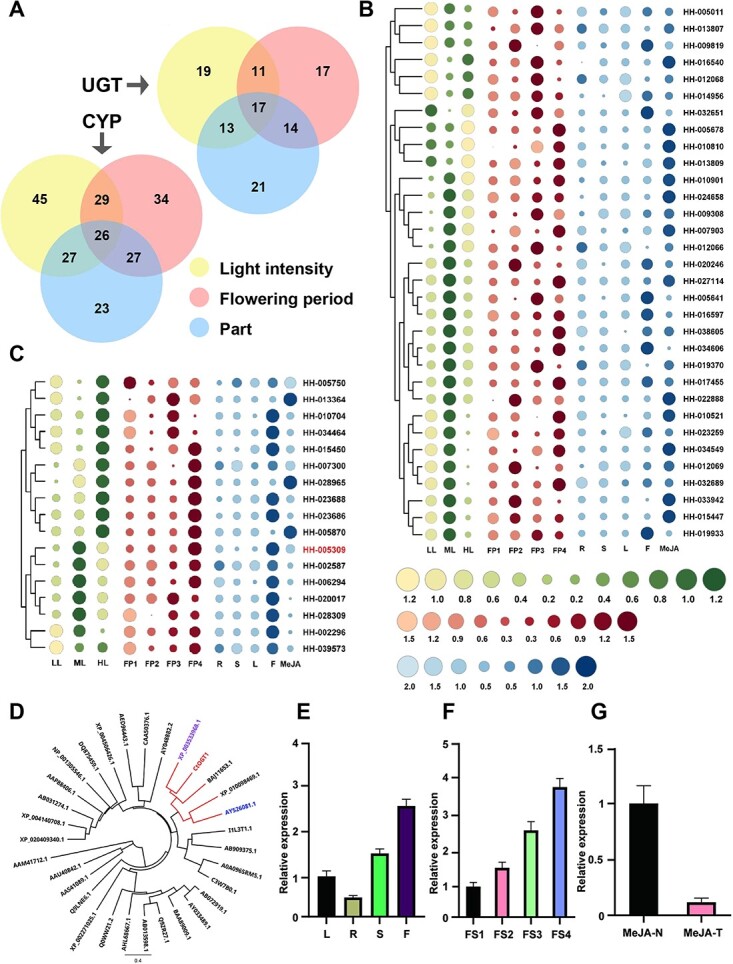
Expression pattern of 26 *CYPs* and 17 *UGTs* related to flavonoids biosynthesis in safflower, and evolutionary and expression analysis of *CtOGT1*. (A) Number of *CYPs* and *UGTs* in each expression analysis. The expression pattern of *CYPs* (B) and *UGTs* (C) at different flowering stages, under different light intensity treatments and under MeJA treatment. (D) Evolutionary relationship of *CtOGT1* with other 30 OGTs. The ML method was used to construct this tree with 1000 replicate bootstrap support. The tree was rooted with AAM41712.1. GenBank IDs of the proteins used and their species names: AAM41712.1, *Xanthomonas campestris pv. Campestris*; AAU40842.1, *Bacillus licheniformis*; AAS41089.1, *Bacillus cereus*; Q9LNE6.1, *Arabidopsis thaliana*; XP_002271025.1, *Vitis vinifera*; Q0WW21.1, *A. thaliana*; AHL68667.1, *Vitis amurensis*; AB013598.1, *Glandularia* × *hybrida*; Q9ZR27.1, *Perilla frutescens*; BAA89009.1, *Petunia* × *hybrida*; AY033489.1, *Solanum sogarandinum*; AB072919.1, *Nicotiana tabacum*; C3W7B0.1, *Oryza sativa* Indica Group; A0A096SRM5.1, *Zea mays*; AB909375.1, *Fagopyrum esculentum*; I1L3T1.1, *Glycine max*; AY526081.1, *Beta vulgaris*; XP_010098469.1, *Morus notabilis*; ABJ11653.1, *Pseudomonas aeruginosa*; XP_003533968.1, *G. max*; AY048882.1, *Citrus maxima*; CAA50376.1, *Petunia* × *hybrida*; AED96443.1, *A. thaliana*; XP_004506426.1, *Cicer arietinum*; DQ875459.1, *Medicago truncatula*; XP_001305546.1, *Trichomonas vaginalis*; AAP88406.1, *Allium cepa*; AB031274.1, *Scutellaria baicalensis*; XP_004140708.1, *Cucumis sativus*; XP_020409340.1, *Prunus persica*. **E** Expression in different tissues. L, leaf; R, root; S, stem; F, flower. **F** Expression during the four stage of flower development. FP1, the first stage of flower development; FP2, the second stage of flower development. FP3, the third stage of flower development; FP4, the fourth stage of flower development. **G** Expression under MeJA treatment. MeJA-N, no MeJA treatment; MeJA-T, MeJA treatment.

The spatially or temporally specific production of secondary metabolites of plants is generally associated with a set of spatially or temporally specifically biosynthetic pathway genes. Previous research has demonstrated that the accumulation of HSYA and kaempferol exhibit an initial increase followed by a decrease in response to rising light intensity. The content of flavonoid glycosides in safflower reaches its highest level on the third to fourth day of flowering when responding to different flowering time. Based on the accumulation patterns exhibited by these flavonoid glycosides, we screened for genes demonstrating similar expression trends. The genes screened under three conditions underwent Venn diagram analysis, which ultimately identified 26 *CYP* genes and 17 *UGT* genes (Fig. 4A–C).

The CYP and UGT genes identified in safflower were subjected to phylogenetic analysis alongside reported homologous genes with known functions, as shown in Fig. S18. The results demonstrated that the 26 CYP genes exhibited low similarity with their 47 homologs (Fig. S18A). In the phylogenetic tree of UGTs with 30 homologs, HH-005309 was clustered with BAJ11653.1 and XP_003533968 as a branch (Fig. S18B). Furthermore, HH-005309 was more closely related to XP_003533968 from *Glycine max*, which is a member of the 7-*O* glycosyltransferase family (Fig. 4D). Consequently, HH-005309 was named as CtOGT1. Expression analysis indicated that *CtOGT1* exhibited constitutive expression, predominantly in flowers. The highest expression levels were observed during the late stages of flower development. We also observed that MeJA treatment could suppress the expression of *CtOGT1* (Fig. 4E–G).

### The function verification of CtOGT1

**Figure 5:**
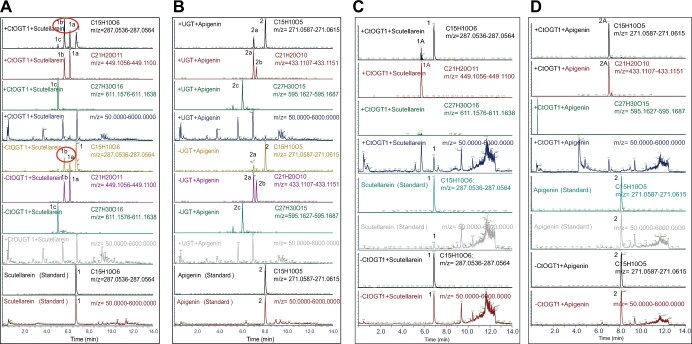
Function verification of CtOGT1 by *N*. *benthamiana* agroinfiltration and prokaryotic expression using scutellarin and apigenin as the substrates. (A) CtOGT1 function verification in *N*. *benthamiana* using scutellarin as substrate. -CtOGT1 means that Agrobacterium not containing this gene was not injected, and + CtOGT1 means that Agrobacterium containing this gene was injected. +scutellarin indicates that the substrate was injected. We tested the standard of scutellarin as a control. According to the molecular weight and MS2, we can know that 1a was one glycoside added to scutellarin, 1b was also one glycoside added to scutellarin, and 1c was two glycosides added to scutellarin. The red circle in the figure shows that when CtOGT1 was transformed, the conversion rate of the product was improved. (B) CtOGT1 function verification in *N*. *benthamiana* using apigenin as substrate for CtOGT1 function verification. +Apigenin indicates that the substrate was injected. We tested apigenin’s standards as controls. According to the molecular weight and MS2, we can know that 1a was one glycoside added to apigenin, 1b is also one glycoside added to apigenin, and 1c was two glycosides added to apigenin. The red circle in the figure shows that when CtOGT1 was added, the conversion rate of the product is improved. (C) CtOGT1 function verification by prokaryotic expression using scutellarin as the substrate. +CtOGT1 + scutellarin mean that there was both the CtOGT1 and scutellarin. -CtOGT1 + scutellarin mean that there was no CtOGT1, but scutellarin. We also detected the standard of scutellarin. Based on the molecular weight and MS2, we can see that 1 was scutellarin, 1A is scutellarin-7O glycoside, and only one product was generated. (D) CtOGT1 function verification by prokaryotic expression using apigenin as the substrate. +CtOGT1 + apigenin means that there was both the CtOGT1 gene and apigenin. -CtOGT1 + apigenin means that CtOGT1 was no CtOGT1, but apigenin. Based on the molecular weight and MS2, we can know that 1 was apigenin, 1A was apigenin-7O glycoside, and only one product was generated.

Agrobacterium infiltration of *N. benthamiana* is an extensively studied method for validating the function of genes [ 26]. In our study, we first used this system to identify the function of CtOGT1. We incorporated scutellarein (Fig. 5A) and apigenin (Fig. 5B) as substrates and observed that the conversion rates of the flavonoid glycosides were higher when both the substrates and CtOGT1 were included, compared to when only the substrates were added, which indicated that CtOGT1 had the activity of glycosyltransferase. However, we detected flavonoid glycosides in the leaves of *N. benthamiana* even without adding CtOGT1, suggesting the presence of endogenous GT in the plant’s leaves.

Therefore, we used a prokaryotic expression system, which is widely used in identifying GT [27, 28]. Initially, we tested the induction conditions and demonstrated that CtOGT1 could be successfully induced and was detectable in the supernatant. We evaluated the activity of CtOGT1 against scutellarein and apigenin using crude enzyme solution and the results indicated that only one new product was generated. To determine the glycosylation position, we compared this product with the standards for scutellarein glycosides and apigenin glycosides. The analysis revealed that glycosylation occurred at the hydroxyl group in the 7th position of the flavonoids, as the new products exhibited the same peak times as scutellarein 7-*O* glycosides and apigenin 7-*O* glycosides. Our results indicated that the enzyme could add only one glycoside to the flavonoids, specifically scutellarein and apigenin (Fig. 5C, D).

### Kinetic parameter analysis of CtOGT1

**Figure 6:**
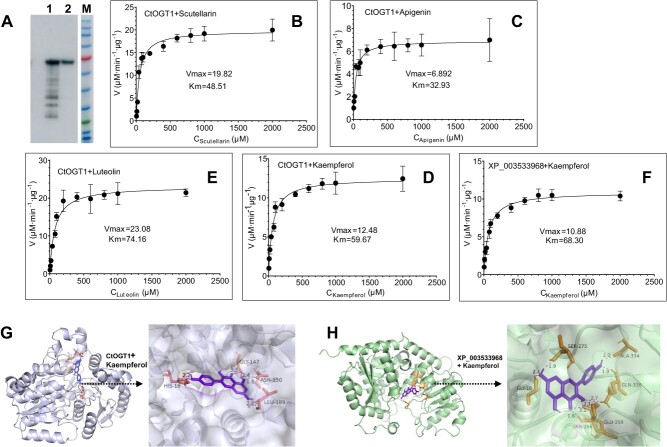
Kinetic parameter analysis of CtOGT1 and molecular docking of CtOGT1 with kaempferol. (A) Western blot for CtOGT1, 1 represents western blot for the total protein of *E. coli* transformed with *CtOGT1*, 2 western blot for the purified protein of CtOGT1. (B) Kinetic parameter analysis of CtOGT1 with scutellarin as substrate. (C) Kinetic parameter analysis of CtOGT1 with apigenin as substrate. (D) Kinetic parameter analysis of CtOGT1 with luteolin as substrate. (E) Kinetic parameter analysis of CtOGT1 with kaempferol as substrate. (F) Kinetic parameter analysis of XP_003533968 with kaempferol as a substrate. (G) The molecular docking of CtOGT1 with kaempferol. (H) The molecular docking of XP_003533968 with kaempferol. Overall and partial drawings are presented. The position and distance of hydrogen bonds were also demonstrated. The position of the red circle is the position of the 7-OH of the flavonoids.

We first purified CtOGT1 and evaluated the purification effectiveness using Western blot analysis (Fig. 6A). The purified proteins were then utilized to assess the kinetic parameters of CtOGT1 on various substrates, such as scutellarein (Fig. 6B), apigenin (Fig. 6C), luteolin (Fig. 6D), and kaempferol (Fig. 6E). The results indicated that the Km values were 48.51 for scutellarein, 32.93 for apigenin, 74.16 for luteolin, and 59.67 for kaempferol. And the corresponding Vmax values were 19.82, 6.892, 23.08, and 12.48 μM·min^−1^ μg^−1^, respectively.

Additionally, we cloned *XP_003533968* from *G. max*, which is also known to exhibit activity against the 7-OH position of flavonoids [29], and compared its enzymatic activity against that of CtOGT1 using kaempferol as the substrate. The results revealed that the Km of XP_003533968 for kaempferol was 68.30, with a Vmax of 10.88 μM·min^−1^ μg^−1^ (Fig. 6F). This indicates that CtOGT1 exhibits higher affinity and sensitivity toward kaempferol. To further investigate this finding, we performed molecular docking studies using AlphaFold to predict the structures of the two proteins and AutoDock for the docking simulations (Fig. 6G, H). The results indicated that the substrates were centrally located within both enzymes. CtOGT1 formed only one hydrogen bond with hydroxyl group in the 7th position of kaempferol, whereas the XP_003533968 protein formed two hydrogen bonds at the same site. This suggests that CtOGT1 provides more spatial accommodation for glycoside binding at this position. Additionally, the hydrogen bond distance for CtOGT1 was measured at 2.1 Å, closer than the 2.4 Å observed for XP_003533968, implying a lower Km value for CtOGT1. The molecular docking results of CtOGT1 with scutellarein, apigenin, and luteolin are presented in Fig. S20.

## Discussion

Safflower is a significant economic crop with flowers abundant in flavonoid glycosides. But the biosynthesis of chalcone glycosides, which are unique to safflower, is still unclear, and the proposed biosynthesis pathways are currently contentious. For example, this study hypothesizes that UGT catalyses the formation of HSYA from intermediate compounds, while Wu *et al*. [30] have suggested that HSYA is formed by the combined catalysis of intermediate compounds by both CYP and UGT. In both hypotheses, CYP and UGT are key genes that catalyse the formation of safflower flavonoid glycosides and influence glycoside diversity. Therefore, this study combines whole-genome and multi-omics analyses, along with phenotypic screening in different tissues, to identify the key genes involved in the biosynthesis of safflower flavonoid glycosides.

CYPs are involved in a range of functions, including the formation of membrane sterols, phytohormones, signal molecules, UV protectants, and volatile organic compounds that facilitate biotic and abiotic interactions, underscoring their significant impact on plant physiological processes [31]. A total of 264 CYP genes were identified in the safflower genome, grouped into 44 families and categorized into 9 clans; however, no genes were assigned to clan 727. It is a common phenomenon that the number and classification of CYPs differs from one plant species to another. For example, research found that 226 *CYP* genes identified from *Dendrobium nobile* were divided into eight clans [32], while 257 *CYPs* identified from *Dendrobium chrysotoxum* were divided into nine clans [32], 136 CYP genes identified by Ankur et al from *Aquilaria agallocha* genome and were assigned them into 8 clans and 38 families [33]. Similar to most angiosperms, the four multifamily clans (71, 72, 85, and 86) were the largest, representing 96.6% of the total cytochrome P450 (CYP) genes in safflower. Plant CYPs are divided into two main categories: A-type, which includes clan 71, and non-A-type, which encompasses all other clans [34]. The safflower CYP genes, categorized into A-type and non-A-type, show distinct differences in gene structure and conserved motifs [35]. Non-A-type CYPs exhibit greater variability in both aspects compared to A-type CYPs. However, four motifs are conserved in both types: the K-helix motif (motif 1), the heme-binding motif (motif 2), the I-helix motif (motif 3), the PERF motif (motif 4), and the proline-rich region (motif 5). These motifs are crucial for the catalytic function of CYP enzymes in safflower. The heme-binding motif enables CYP proteins to efficiently catalyze the hydroxylation of flavonoid compounds. The K-helix motif ensures the precise positioning of flavonoids within the enzyme, while the I-helix motif facilitates oxygen activation, thereby enabling the hydroxylation process [36] [37]. Furthermore, most non-A-type CYPs lack four specific motifs (motifs 5, 11, 13, and 15) at the C-terminal and one motif (motif 16) at the N-terminal. These five motifs were found commonly in A-type CYPs and are recognized as serving an important role in A-type CYPs. In summary, the structural diversity of safflower CYPs is likely responsible for the development of various physiological activities, which enables the biosynthesis of a broad spectrum of active flavonoids. The CYP gene family is extensive and functionally diverse across organisms, and classifying CYP genes into specific families aids in understanding their roles within the plant metabolic network. Among these clans, clans 97, 74, 710, and 51 are associated with primary metabolism in plants [38]. Genes in clans 97, 72, 711, 85, 74, 51, and 71 are involved in the plant’s response to environmental stress [39]. Genes in clans 86, 97, 72, and 71 can influence the plant’s response to, synthesis, or metabolism of hormones [40]. Members of clans 51, 71, 85, and 86 are involved in secondary metabolism, which can affect the synthesis of flavonoids, terpenoids, and alkaloids [41]. For example, CYP76 and CYP706 are known to catalyze terpenoid biosynthesis, while the CYP75 and CYP93 families typically catalyze flavonoid biosynthesis [42]. Given safflower’s specific biosynthesis of flavonoid glycosides, a detailed and comprehensive dataset of the CYP gene family is essential to establish a foundation for the identification and screening of functional genes [36].

GTs catalyze the transfer of glycosyl molecules from donor molecules to acceptors, with UGT representing the most extensive gene family within this group [43]. UGTs serve multiple roles, including the regulation of growth and development, defence against pathogens and abiotic stress, and adaptation to diverse environments [44]. Based on phylogenetic relationships, UGTs can currently be categorized into 18 groups. Fourteen groups, numbered A–N, are identified in *Arabidopsis thaliana* [45], while four additional groups, numbered O-R, have been subsequently discovered in *Malus* × *domestica* [46], *Zea mays* [47], and *Citrus sinensis* [48]. Significant variations exist in the grouping and number of UGTs across different species. For example, 152 UGT genes, clustered into 13 groups, were identified in the *Morella rubra* genome [49]. Additionally, 157 genes in the *Gossypium raimondii* genome were assigned into 15 groups [44]. In our study, 140 UGT genes were identified in the safflower genome and divided into 15 groups. This classification includes 13 groups previously discovered in Arabidopsis and two newly identified groups. Notably, no genes were classified into groups F, P, and R. As reported in *Arabidopsis*, *Brassica species*, maize, and soybean, UGT proteins related to polyphenolic and flavonoid synthesis are consistently enriched in subgroups A, E, F, L, and M. Proteins associated with terpene metabolism are enriched in subgroups D and M, while those related to plant hormones are enriched in subgroups H, L, K, and O. Additionally, groups G–N are primarily involved in enzymes related to plant secondary metabolism, carbohydrate metabolism, and lipid metabolism [50]. The number and distribution of conserved motif can uncover structural similarities and differences among gene family members and is an important foundation for phylogenetic classification in safflower UGT gene family, 20 conserved motifs were analyzed, eight motifs (motif 1, 2, 3, 4, 5, 6, 10, and 11) were present in all groups, indicating their potential importance in the UGT gene family composition. Conversely, the remaining 14 motifs may play a significant role in defining subgroups. The conserved motifs in UGT proteins are crucial for maintaining the enzyme’s overall structural stability and its binding with flavonoid substrates or sugar donors. For instance, the D/EX7E motif (Motif 11, D/ExxxxxxE) facilitates the transfer of glycosyl groups to flavonoid compounds by interacting with both the substrate and the sugar donor [51]. Alterations in exon/intron structure can lead to variations in gene or protein functions. In this study, it was observed that most UGT genes within the same group exhibit similar exon/intron structures and numbers, a pattern consistent with other plants such as *Nicotiana tabacum* [50], *Medicago sativa* L. [25], and wheat [52]. Like most plants, flavonoids in safflower primarily exist in the form of glycosides. The diversity of these flavonoid glycosides is heavily influenced by the glycosylation positions (e.g., hydroxyl or carbonyl groups) and the types of sugar donors (e.g., glucose, galactose, or rhamnose). These factors are crucial for categorizing UGT groups and understanding the classification of the UGT gene family in safflower, which can save time and resources in screening for target genes. This detailed understanding of the UGT family facilitates the identification of specific enzymes involved in the biosynthesis of flavonoid glycosides in safflower [53].

Our previous studies have found that the content of chalcone glycosides significantly increases following MeJA treatment; the flavonoid glycosides rise during the first to third or fourth days of flowering; and their content fluctuates with changes in light intensity. In the promoter analysis of CYPs and UGTs, a large number of *cis-*acting elements associated with light, growth, hormones, and stress were identified, suggesting that both CYP and UGT genes are responsive to these factors, which further confirmed that CYP and UGT are crucial genes in the biosynthesis of safflower flavonoid glycosides. Based on the gene expression patterns of CYPs and UGTs under different conditions, these genes were categorized into different groups. At the same time, phenotypic and metabolic analyses of roots, stems, and leaves confirm that the distribution of flavonoid glycosides in safflower exhibits clear tissue specificity. Additionally, chalcone glycosides are synthesized exclusively in the flowers of safflower. Therefore, this study integrated analyses of gene expression and flavonoid accumulation patterns for candidate gene screening. However, evolutionary analysis indicated that the candidate safflower CYP genes did not cluster with previously identified CYP genes, suggesting that these candidate CYPs may not be functional. In the evolutionary analysis of UGTs, one UGT (HH-005309, named CtOGT1) closely clustered with two previously identified UGT genes, indicating that this gene likely belongs to the 7-*O*-GT.

Most glycosyltransferases are characterized by a broad substrate promiscuity, which enables them to generate diverse glycosylated products from numerous substrates. Most GTs exhibit broad substrate promiscuity, enabling them to glycosylate various positions on diverse substrates. For example, the O-GT from *Tripterygium wilfordii*, TwUGT2, specifically targets the 3-OH and 7-OH groups of flavonoids [54]; it is also reported that 43 OGT candidate genes were screened from *Glycyrrhiza uralensis*, and 11 of them were characterized, including isoflavone 7-*O*GTs, flavonol 3-*O*GTs, and promiscuous OGTs [55]. However, the gene we screened could only add one glucoside to substrates. It was found that all three substrates can be glycosylated. It is possible that other GT are involved in the addition of glucosides at different positions. Therefore, it is crucial to thoroughly investigate the specificity of O-GTs toward different substrates and positions to fully understand their biological activities. Our results indicated that the prokaryotic expression system is an effective method for studying GT, using either crude or purified enzymes. For large-scale screening, activity analysis can be directly performed using crude enzyme solutions. Furthermore, we conducted molecular docking for three other substrates: scutellarin, apigenin, and luteolin (Fig. S2) and identified potential active sites at HIS16, LEU-189, ASN-150, and GLY147. Future research may explore mutations to optimize enzyme activity.

Another interesting finding from this experiment was that MeJA inhibited the expression of the UGT gene, despite previous studies indicating that MeJA promotes the biosynthesis of flavonoids, particularly quinone–chalcone components such as HSYA, which are *C*-glucosides [56]. It is possible that there are differences in the regulatory patterns of *C*-glucosides and *O*-glucosides in safflower. Prior research by Guo indicated that three UGTs are closely associated with *O*-glucosides, although their functions have yet to be confirmed. Furthermore, the expression patterns of these three UGTs revealed that all were suppressed in response to MeJA induction [57]. Therefore, further research into the regulatory modes of *O*-glucoside and *C*-glucoside biosynthesis is essential to enhance our understanding of their differences in safflower. A thorough investigation of these mechanisms could provide insights into the complex pathways that promote flavonoid biosynthesis and optimize their biological activities.

The enzymes CYP and UGT play critical roles in the biosynthesis, distribution, and diversity of flavonoid glycosides in safflower. The study employs an integrated approach combining whole-genome and multi-omics analyses to identify key genes responsible for the biosynthesis of safflower flavonoid glycosides. It includes gene family analysis of 264 CYP and 140 UGT gene family members in the safflower genome, examining their phylogenetic relationships, conserved motifs, gene structures, cis-acting elements, and chromosome mapping. The analysis yielded extensive and detailed gene family data. Candidate genes were selected based on the expression patterns of safflower CYP and UGT genes across various developmental stages, light conditions, tissues, and MeJA treatments. Subsequently, a highly active UGT gene was identified through evolutionary analysis with reported genes. Functional validation and enzyme activity studies were then conducted. In summary, this study successfully identified a functional gene through a comprehensive gene selection strategy that combined whole-genome and multi-omics approaches. It provided valuable research ideas and a data foundation for the biosynthesis and pathway analysis of safflower flavonoids, thereby establishing a foundation for further analysis and functional interpretation.

## Materials and methods

### Identification of CYPs and UGTs in the safflower genome

The safflower genome was derived from our previous study [12]. Briefly, to identify the CYP and UGT gene families in safflower, the Arabidopsis Information Resource (http://www.arabidopsis.org/) was consulted to get the sequences for CYPs and UGTs from *Arabidopsis*. Additionally, hidden markov models (HMMs) for the conserved domains of CYPs (PF00067) and UGTs (PF00201) were obtained from PFAM 35.0 (http://pfam.xfam.org/). These models were utilized as queries in hmmsearch against safflower protein sequences using HMMER 3.2.1, applying an e-value threshold of 0.1 (http://hmmer.org/). A local BLASTP search was performed against the safflower protein database using amino acid sequences from *A. thaliana* as query sequences. The search was conducted with an e-value threshold of 1e-5. The identified CYP sequences, with protein lengths ranging from 300 to 650 amino acids, along with all UGT sequences, were subsequently validated using the NCBI Conserved Domain Database tool.

The physical and chemical parameters of CYPs and UGTs, such as amino acid count, molecular weight, theoretical isoelectric point, instability index, aliphatic index, and grand average of hydropathicity, were calculated using TBtools software [58]. Subsequently, subcellular localization predictions for these sequences were conducted via the online platform Plant-mPLoc (http://www.csbio.sjtu.edu.cn/bioinf/plant-multi/#).

### Phylogenetic analysis, conserved motifs, gene structures, and chromosomal mapping

The protein sequences of CYPs and UGTs were aligned utilizing the MUSCLE algorithm incorporated in the MEGA software (https://www.megasoftware.net/), respectively. Regions with very low similarity were automatically trimmed using the trimAl function of the TBtools software [59]. Subsequently, phylogenetic trees were generated from the trimmed alignments employing the maximum-likelihood (ML) approach, with bootstrap values calculated over 5000 replicates. Finally, the constructed phylogenetic trees were annotated and visually presented using the online platform EVOLVIEW (https://evolgenius.info/evolview-v2/#login) [60].

The conserved motifs within CYPs and UGTs were detected using MEME (https://meme-suite.org/meme/tools/meme), an online tool specifically designed for motif identification [61]. The parameters were configured as follows: the number of motifs to identify was set to 20, with a minimum motif width of 6 and a maximum motif width of 50. Gene structures and chromosomal locations of CYPs and UGTs were ascertained using the GFF files of safflower genomes. The conserved motifs, gene structures, and chromosomal locations were visualized and modified with the assistance of TBtools.

### *Cis*-acting element analysis in promoters

To analyse *cis*-acting elements, we retrieved the 2000 base pair (bp) upstream sequences of the initiation codon (ATG) for the CYP and UGT genes from the safflower genome. These promoter sequences were analyzed using the PlantCARE database (http://bioinformatics.psb.ugent.be/webtools/plantcare/html/) to predict potential *cis*-acting elements. We focused on elements associated with development, stress, hormone, and light responses, which were then retained for further visualization and analysis.

### Flavonoid composition analysis of safflower

Safflower plants were cultivated at the Medicinal Botanical Garden located on the Wenjiang campus of Chengdu University of Traditional Chinese Medicine in Chengdu, China. Safflower tissues, including roots, stems, and leaves were cross-sectioned and allowed to exude a liquid RS for 30 seconds. This RS was gently aspirated and dissolved in methanol. The samples, along with HSYA standards, were analyzed using ultra-performance liquid chromatography (UPLC) and mass spectrometry (MS). For the metabolic analysis, the safflower tissues were freeze-dried under vacuum, then ground into powder at 30 Hz for 1.5 minutes using a grinder (MM 400, Retsch). Next, 50 mg of the powdered sample was weighed, and 1200 μL of pre-cooled (−20°C) 70% methanolic aqueous solution was added at a ratio of 1200 μL per 50 mg of sample. The mixture was vortexed every 30 minutes for 30 seconds, repeated six times. After centrifugation at 12000 rpm for 3 minutes, the supernatant was carefully aspirated and filtered through a 0.22-μm microporous membrane. The filtered solution was then stored in an injection vial for subsequent UPLC-MS/MS analysis. The safflower roots, stems, and leaves were collected after the safflower bolted, with each sample collected from three different plants. For the metabolic analysis of safflower roots, stems, and leaves, one replicate was used, and the experimental procedures followed those described in our previous study [62]. The metabolomic data of safflower flowers were derived from our previous analyses, with each sample coming from 10 different flowers and three replicates were performed [56].

## Transcriptome analysis of safflower

During the flowering period, four developmental stages were selected. The first stage corresponds to the first day after anthesis (DAA) (FP1), the second to two DAA (FP2), the third to three DAA (FP3), and the fourth to four DAA (FP4). Also, the roots, stems, leaves, and flower were collected. MeJA treatments were conducted based on our previous research [56]. Briefly, a 100-μM solution of MeJA (Sigma-Aldrich, Switzerland) was sprayed onto flowers at three DAA, and the flowers were then enclosed in clear plastic bags. After 6 hours of treatment, the flower samples were collected. Three samples (each with three repetitions) were collected for each flower sample in the above experiment. The root, stem, and leaf samples constituted one replicate, and all samples were immediately frozen in liquid nitrogen to preserve them for RNA extraction and subsequent expression analysis. Furthermore, transcriptomes of safflower treated with MeJA and different light intensities (low light intensity, LL; medium light intensity, ML; high light intensity, HL) (LL: 10000 Lux, ML: 20000 Lux, HL: 40000 Lux) were obtained from our previous study [56, 63]. In this study, we collected expression data for safflower CYPs and UGTs from these transcriptomic datasets. These data were then converted into FPKM (fragments per kilobase of exon per million fragments mapped) values for further analysis. The genes were grouped according to their expression levels, and expression heatmaps were generated using the Chiplot tool (https://www.chiplot.online/). Based on the expression patterns of these genes, the online software (http://www.cloud.biomicroclass.com) were used to classify them. Perform Venn analysis on the genes selected from the needed clusters (https://jvenn.toulouse.inrae.fr/app/example.html) [64]. Finally, conduct evolutionary analysis on the genes that were commonly selected under three conditions to identify the candidate functional genes.

### Expression pattern analysis and isolation of the candidate genes

Gene expression in four organs, flowers at different developmental stages, and MeJA-treated samples was determined using real-time PCR. Samples were collected, quickly frozen in liquid nitrogen, and total RNA was isolated using the TRIzol Chaperone Kit (Tiandz, Beijing, China). cDNA was synthesized from the isolated RNA through reverse transcription. Real-time PCR was then performed on these cDNA samples with gene-specific primers listed in Table S7. The procedure followed established protocols, and agarose gel electrophoresis confirmed the specificity of the PCR reactions. All experiments were conducted in triplicate.

### Construction of transgenic vectors and agroinfiltration of *N. Benthamiana*

The CDS sequence of *CtOGT1* (*HH-005309*) was extracted from the genome and specifically amplified using the primers listed in the Table S7. *CtOGT1* was then cloned into the *pMD19-T* vector (Takara, Beijing, China) and sequenced to confirm its accuracy. The *CtOGT1* gene was initially cloned into the *pDONR207* vector using the BP reaction, utilizing the Gateway BP Clonase II Enzyme Mix (Thermo Fisher, MA, USA). Subsequently, the *CtOGT1* gene was introduced into the *pEAQ11* vector using the LR reaction, with primers listed in Table S7. The agroinfiltration process of *N. benthamiana* involves several key steps. Initially, bacteria from fresh colonies or glycerol stock were cultured in 10 ml of LB medium with selective antibiotics. This culture was centrifuged for 15 minutes at 2000 rpm, after which the supernatant was discarded. The cells were then resuspended in 15 ml of agroinfiltration solution and subjected to a second centrifugation at 2500 rpm for 15 minutes.

The supernatant was removed, and the cells were resuspended in 10 mL of agroinfiltration solution containing 200 μM acetosyringone. After a 2-hour incubation at room temperature with gentle shaking in the dark, the optical density (OD_600_) of each culture was measured and adjusted to 0.2 in a total volume of 10 ml with the same agroinfiltration solution. Using a 1 ml syringe, 0.5–1 ml of the mixture was infiltrated into the underside of *N. benthamiana* leaves. Leaves were typically harvested 4–6 days later for composition analysis.

### Extraction and detection of *N. benthamiana* leaves components

The extraction method for leaves used in this study was based on previous research [52]. Briefly, a 10-mg sample of freeze-dried leaves was accurately weighed using a microbalance and placed into 2 ml screw-top microcentrifuge tubes, along with two tungsten beads. These samples were homogenized using a tissue lyser at 1000 rpm for 1 minute and briefly spun down at 20000 g. Next, 400 μl of 50% methanol was added to each sample, and they were placed in an ultrasonic bath set to 50% amplitude at 4°C for a total of 15 minutes, with 20-second on and 40-second off intervals. Each sample was then transferred to a fresh 1.5 ml microcentrifuge tube on ice, avoiding carrying over any leaf material when possible. The samples were further processed using filter tubes and spun at 12500 g for 30 s. A 50 μl volume of the resultant filtrate was transferred to an autosampler glass insert vial containing an additional 50 μl of 50% methanol.

Data acquisition was performed using a Thermo Scientific™ UltiMate 3000 UHPLC and Q Exactive benchtop Orbitrap mass spectrometer. The column utilized was an Eclipse Plus C18 (150 mm × 3.0 mm, 1.8 μm) from Agilent, USA. The mobile phases comprised pure water with 0.1% formic acid (A) and acetonitrile (B). The elution gradient was programmed as follows: from 0 to 25 minutes, phase B increased from 5% to 95%, with a flow rate of 0.2 mL/min. The absorption intensity was recorded at a wavelength of 280 nm, and ESI-MS spectra were obtained in positive ion mode, generating [M + H] + or [M + Na] + ions, using dry gas N_2_ at 800 L/h with a temperature of 450°C, sprayer pressure of 6 bar, and capillary voltage of 3 kV. The mass scanning range m/z 100 to 800 was used, and the molecular weight of the target compound was determined by extracted ion chromatograms (EICs).

### Expression, purification and WB analysis of recombinant proteins

The prokaryotic expression of the *CtOGT1* gene relied on the *pET32a* vector (Thermo Fisher, USA), with primers provided in Supplementary Table 1. The recombinant vectors, *pET32a-CtOGT1*, were transformed into the *BL21* (DE3) strain. *BL21* cells were cultured in LB medium containing 50 μg/ml ampicillin until the OD_600_ reached 0.5. Then, the cell induction was performed with 120 μM IPTG and shaking at 16°C for 16 hours. After incubation, they were harvested and resuspended in 30 ml of lysis buffer (25 mM HEPES pH 8, 500 mM NaCl, 5 mM imidazole) for sonication on ice. Following sonication, the mixture was centrifuged to remove the supernatant. The resulting cell pellet was used for detection of enzyme activity.

The enzyme was purified to assess its kinetic parameters. To achieve this, the supernatant was filtered through a 0.45-μM filter prior to batch binding. Ni-NTA resin (Qiagen, Germany) was then added to the filtrate at a ratio of 1.5 ml per liter of cell culture, followed by a 1-hour incubation at 4°C. The mixture was applied to a gravity flow column. After discarding the flow-through, the column was washed with wash buffer (25 mM HEPES pH 8, 100 mM NaCl, 20 mM imidazole). The tagged protein was eluted with elution buffer (25 mM HEPES pH 8, 100 mM NaCl, 250 mM imidazole). Ultimately, a Bradford assay was used for monitoring the concentration. CtOGT1 was concentrated using a 30 kDa Amicon Ultra spin filter, and 10% v/v glycerol was added. After flash freezing, the proteins were stored at −80°C. Western blot analysis of CtOGT1 was performed as described in a previous report [65].

### Detection of prokaryotic expression reaction products

Initially, the glycan transfer activity of CtOGT1 was tested using a prokaryotic expression crude enzyme solution. The reaction products were identified through UHPLC analysis by comparing them with corresponding glycoside standards. The reaction conditions involved using a 1-mM acceptor substrate alongside 2 mM UDP-glucose, 10 μM of purified recombinant protein, and a buffer of 50 mM Na_2_HPO_4_-NaH_2_PO_4_ (pH 8) in a 100-μl reaction system. The reaction was conducted at 25°C for 5 minutes. Once completed, we quickly mixed and homogenized 200 μl of pre-cooled methanol, centrifuged at 12000 rpm for 15 minutes, and then loaded the supernatant into the injection bottle. For UHPLC, the experimental details were as previously described, and the gradient elution process started with 5% to 95% phase B over 0–40 minutes, followed by a 0–25 minute period at a flow rate of 0.2 ml/min. Subsequently, we characterized the products using a Thermo Scientific™ UltiMate 3000 UHPLC and Q Exactive benchtop Orbitrap mass spectrometer (Thermo Fisher, USA). We injected a volume of 3 μl into the ion source, which had a spray voltage of 3.2 kV, an ion source temperature of 350°C, sheath gas flow rate was set at 35 arb, and the auxiliary gas flow rate at 10 arb, and the ion transport tube temperature was maintained at 320°C. For positive ion detection, we employed a full MS/DD-MS2 scanning mode with a first-level resolution of 35 000 and a second-level resolution of 17 500, covering a scanning range of m/z 100 to 1500 and utilizing an impact energy gradient of 20, 40, and 60 eV.

### Determination of enzyme kinetic parameter

To determine the kinetic parameters of the safflower glycosyltransferase recombinant protein catalyzing the glycosylation of kaempferol with two sugars, we established a reaction system using 50 mM Na_2_HPO_4_-NaH_2_PO_4_ (pH 8) buffer with excess UDP-glucose, and a recombinant protein concentration of 1 μM. The acceptor substrate, kaempferol-7-O-glucoside, was added with concentrations ranging from 5 μM to 1200 μM. The reaction was conducted at 25°C for 5 minutes, after which the reaction was quickly quenched by adding 200 μl of pre-cooled methanol and centrifuging at 12000 rpm for 15 minutes. The supernatant was collected and loaded into the injection bottle to quantify the product content using UPLC, with the detection conditions the same as previously described. The experiments were repeated three times. In addition, to determine the kinetic parameters of the safflower glycosyltransferase recombinant protein on the substrate, a similar reaction system was used, with kaempferol-7-*O*-glucoside selected as an intermediate product for the determination of the acceptor substrate. The reaction was carried out with varying concentrations of the substrate (5 μM to 1500 μM) and excess UDP-glucose using the same buffer and protein concentration as before at a temperature of 25°C for 5 minutes. The reaction was quickly quenched with methanol, and the product content was quantified using UHPLC, with detection conditions matching those previously described. The experiments were also repeated three times. These analytical procedures were necessary to accurately determine the kinetic parameters of the safflower glycosyltransferase recombinant protein on the substrate and to select the optimal reaction conditions for further experimentation.

### Molecular docking

The three-dimensional structure of the CtOGT1 protein was constructed using AlphaFold (https://www.alphafold.ebi.ac.uk/), while the 3D structures of the ligands were obtained from PubChem (https://pubchem.ncbi.nlm.nih.gov/). Molecular docking of CtOGT1 with the flavonoids scutellarein, apigenin, luteolin, and kaempferol was performed using AutoDock Tools [66]. The modeling molecular docking results was visualized using PyMOL [67].

## Supplementary Material

Web_Material_uhae261

## Data Availability

The data used in this study are included in the article.
