# Supplementary material for: Genome-wide screen and multi-omics analysis reveal OGT1 participate in the biosynthesis of safflower flavonoid glycosides
Source: Hortic Res. 2024 Sep 16;11(12):uhae261. doi: 10.1093/hr/uhae261 (PMC11632156; doi:10.1093/hr/uhae261)

Supplementary figures

Figure S1 *CYPs* and *UGTs* genes in the biosynthetic pathway of two groups of flavonoids in safflower.

Figure S2 Schematic diagram of conserved motifs in safflower CYPs, with different motifs represented by various colors.

Figure S3 WebLogos of the 20 conserved motifs in safflower CYPs. Each letter in the WebLogos represents an amino acid, with its height indicating the relative frequency of that amino acid.

Figure S4 Schematic diagram of conserved motifs in safflower UGTs, with different motifs represented by various colors.

Figure S5 WebLogos of the 20 conserved motifs in safflower Each letter in the WebLogos represents an amino acid, with its height indicating the relative frequency of that amino acid.

Figure S6 Gene structures' schematic diagram in safflower *CYPs*. Green and pink rectangles denote untranslated regions (UTR) and coding sequences (CDS), respectively. Lines without rectangles represent introns.

Figure S7 Gene structures' schematic diagram in safflower *UGTs*. Dark and purple rectangles denote UTR and CDS, respectively. Lines without rectangles represent introns.

Figure S8 Gene structures' schematic diagram in safflower *UGTs*. Dark and purple rectangles denote UTR and CDS, respectively. Lines without rectangles represent introns.

Figure S9 Schematic diagram of four categories of *cis*-acting elements in the promoter of safflower *CYPs*.

Figure S10 Heatmap display of the number of each *cis*-acting element in the promoter of safflower *CYPs*.

Figure S11 Number of *cis*-acting elements in the promoter region of safflower *UGTs*. The predicted *cis*-acting elements were classified into development, stress, hormone, and light responsiveness.

Figure S12 Schematic diagram of four categories of *cis*-acting elements in the promoter of safflower *UGTs*.

Figure S13 Heatmap display of the number of each *cis*-acting element in the promoter of safflower *UGTs.*

Figure S14 Expression of *CYPs* in the of safflower at different flowering stages, under different light intensity treatments and under MeJA treatment. Expression levels of safflower *CYPs* at different flowering stages (**A**), under different light intensity treatments (**B**), and at different tissue and under MeJA treatment (**C**) were shown in the form of a heat map. The average expression value for each gene was automatically adjusted and displayed as heatmaps using TBtools. The expression values, which were obtained from our previous research, are detailed in Table S1.

Figure S15 Expression of *UGTs* in the of safflower at different flowering stages and tissue, under different light intensity treatments and under MeJA treatment. Expression levels of safflower *UGTs* at different flowering stages (**A**), under different light intensity treatments (**B**), and at different tissue and under MeJA treatment (**C**) were shown in the form of a heat map. The average expression value for each gene was automatically adjusted and displayed as heatmaps using TBtools. The expression values, which were obtained from our previous research, are detailed in Table S2.

Figure S16 Expression clusters of *CYPs* in safflower at different flowering stages and tissues, under different light intensities treatments and under MeJA treatment. Genes were categorized into six separate clusters and assigned to nine CYP clans according to their expression patterns during various flowering stages (**A**), under different light intensities (**B**), and across different tissues and in response to MeJA treatment (**C**). The expression values, sourced from our prior research, are available in Table S1.

Figure S17 Expression clusters of UGTs in safflower at different flowering stages and tissues, under different light intensities treatments and under MeJA treatment. Genes were categorized into six separate clusters and assigned to nine CYP clans according to their expression patterns during various flowering stages (**A**), under different light intensities (**B**), and across different tissues and in response to MeJA treatment (**C**). The expression values, sourced from our prior research, are available in Table S2.

Figure S18 Phylogenetic analysis of *CYPs* and *UGTs* screened in safflower with homologues of reported functions. The maximum-likelihood method was used to construct this tree with 1000 replicate bootstrap support. **A** The phylogenetic tree of safflower 26 CYPs together with 40 homologues. The tree was rooted with AlI16849.1. GenBank IDs of the proteins used and their species names: AlI16849.1, *Enterobacter sp.*; BAB59004.1, *Perilla frutescens var. crispa*; AAC39454.1, *Eschscholzia californica*; ABB20912.1, *Stevia rebaudiana*; JX162214.1, *Mentha* × *piperita*; JX162212.1, *Ocimum basilicum*; MF363008.1, *Scutellaria baicalensis*; MF363006.1, *Scutellaria baicalensis*; CAA71515.1, *Glycine max*; AAS90162.1, Human immunodeficiency virus 1; O49394.2, *Arabidopsis thaliana*; BAK20464.1, *Eschscholzia californica*; DQ131886.1, *Nicotiana tabacum*; NP189154.1,

Arabidopsis thaliana; BAT21088.1, *Delphinium semibarbatum*; ACR14867.1, *Malus domestica*; NP001237015.1, *Glycine max*; NP196416.1, *Arabidopsis thaliana*; BAX90121.1, *Raphanus sativus*; BAE47006.1, *Vitis vinifera*; XP028111185.1, *Camellia sinensis*; Q9SBQ9.1, *Petunia × hybrida*; BAB87838.1, *Torenia hybrid cultivar*; AAV74195.1, *Sorghum bicolor*; ACD42776.1, *Catharanthus roseus*; P93531.1, *Solanum chacoense*; P93530.1, *Solanum chacoense*; A6YIH8.1, *Hyoscyamus muticus*; Q9XHE6.1, *Mentha × piperita*; Q9XHE7.1, *Mentha × piperita*; Q6WKZ1.1, *Mentha × gracilis*; O81974.1, *Glycine max*; NP001236165.1, *Glycine max*; P98183.2, *Catharanthus roseus*; NP001304582.2, *Glycine max*; CAA71514.1, *Glycine max*; O22307.1, *Lotus japonicus*; MF363004.1, *Scutellaria baicalensis*; MF363005.1; *Scutellaria baicalensis*. **B** The phylogenetic tree of safflower 17 UGTs together with 30 homologues. UGT gene for further studies was indicated in red font. The tree was rooted with AB072919.1. GenBank IDs of the proteins used and their species names: AB072919.1, *Nicotiana tabacum*; C3W7B0.1, *Oryza sativa* Indica Group; A0A096SRM5.1, *Zea mays*; AB909375.1, *Fagopyrum esculentum*; I1L3T1.1, *Glycine max*; XP_010098469.1, *Morus notabilis*; AY526081.1, *Beta vulgaris*; XP_003533968.1, *Glycine max*; ABJ11653.1, *Pseudomonas aeruginosa*; Q0WW21.1, *Arabidopsis thaliana*; AHL68667.1, *Vitis amurensis*; BAA89009.1, *Petunia × hybrida*; AY048882.1, *Citrus maxima*; Q9ZR27.1, *Perilla frutescens*; XP_002271025.1, *Vitis vinifera*; AAP88406.1, *Allium cepa*; DQ875459.1, *Medicago truncatula*; XP_020409340.1, *Prunus persica;* XP_004140708.1, *Cucumis sativus*; XP_004506426.1, *Cicer arietinum*; NP_001305546.1, *Solanum tuberosum*; Q9LNE6.1, *Arabidopsis thaliana*; AB013598.1, *Glandularia × hybrida*; AAU40842.1, *Bacillus licheniformis*; AAS41089.1, *Bacillus cereus*; AAM41712.1, *Xanthomonas campestris pv. campestris*; AY033489.1, *Solanum sogarandinum*; AED96443.1, *Arabidopsis thaliana*; CAA50376.1, *Petunia × hybrida*; AB031274.1, *Scutellaria baicalensis.*

Figure S19 Distribution of the red substance in the cross-section of safflower root, stem and leaf. **A** The flower of safflower. **B** Cross-section of safflower root. **C** Cross-section of safflower stem. **D** Cross-section of safflower leaf. The red substance was marked by an arrow.

Figure S20 Molecular docking of *CtOGT1* with scutellarein, apigenin and luteolin.

Supplementary figure S1

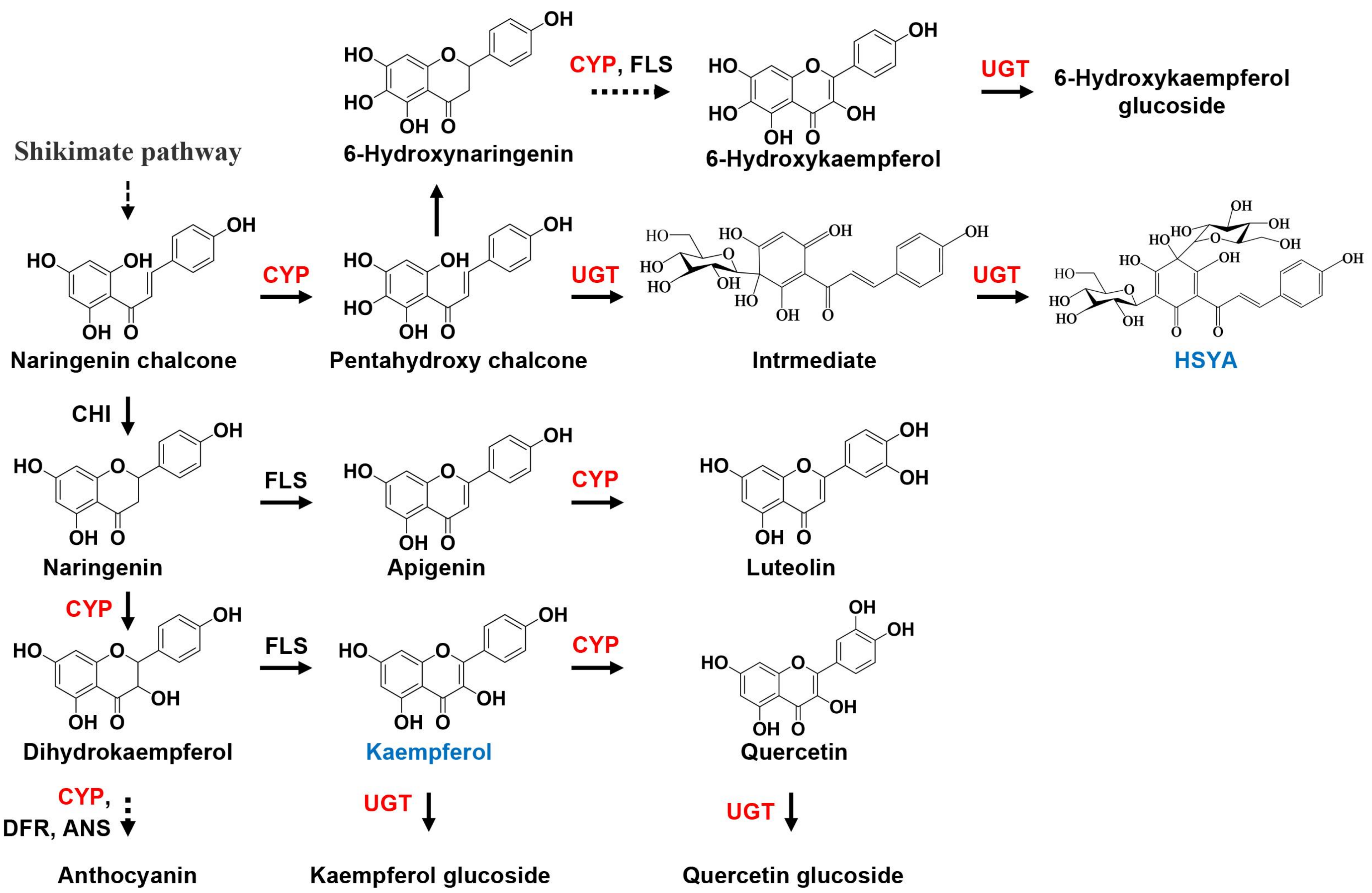

Supplementary figure S2

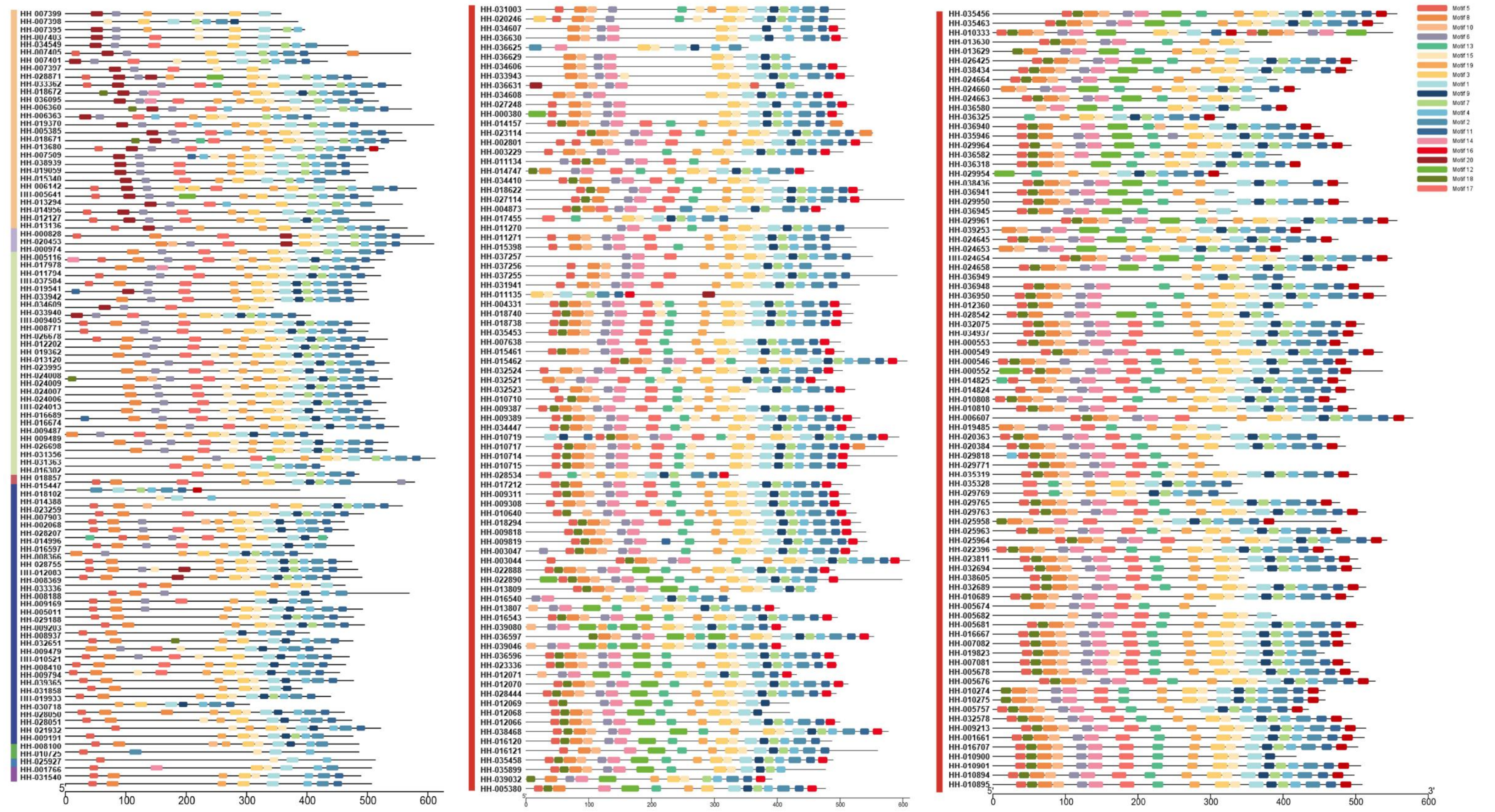

Supplementary figure S3

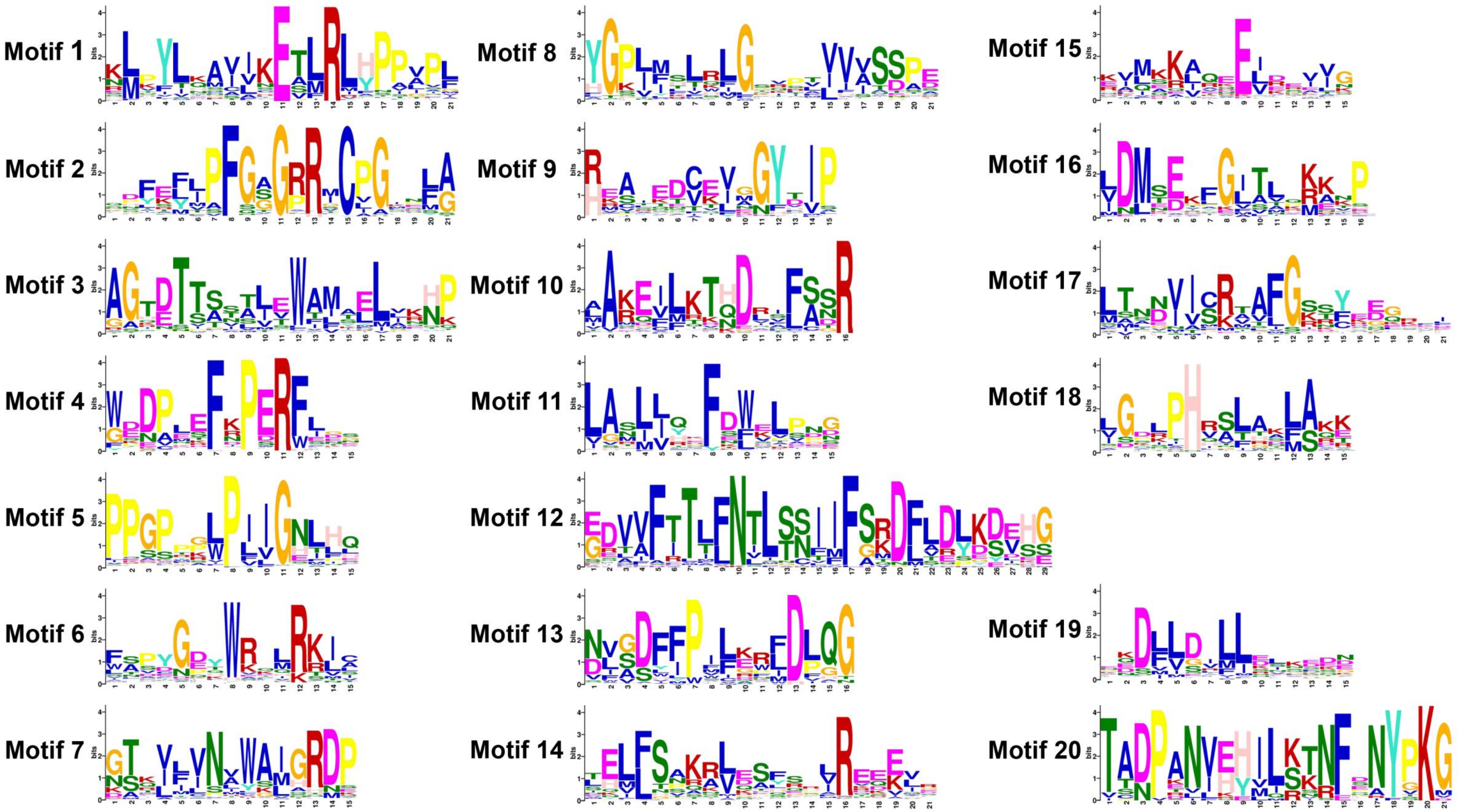

Supplementary figure S4

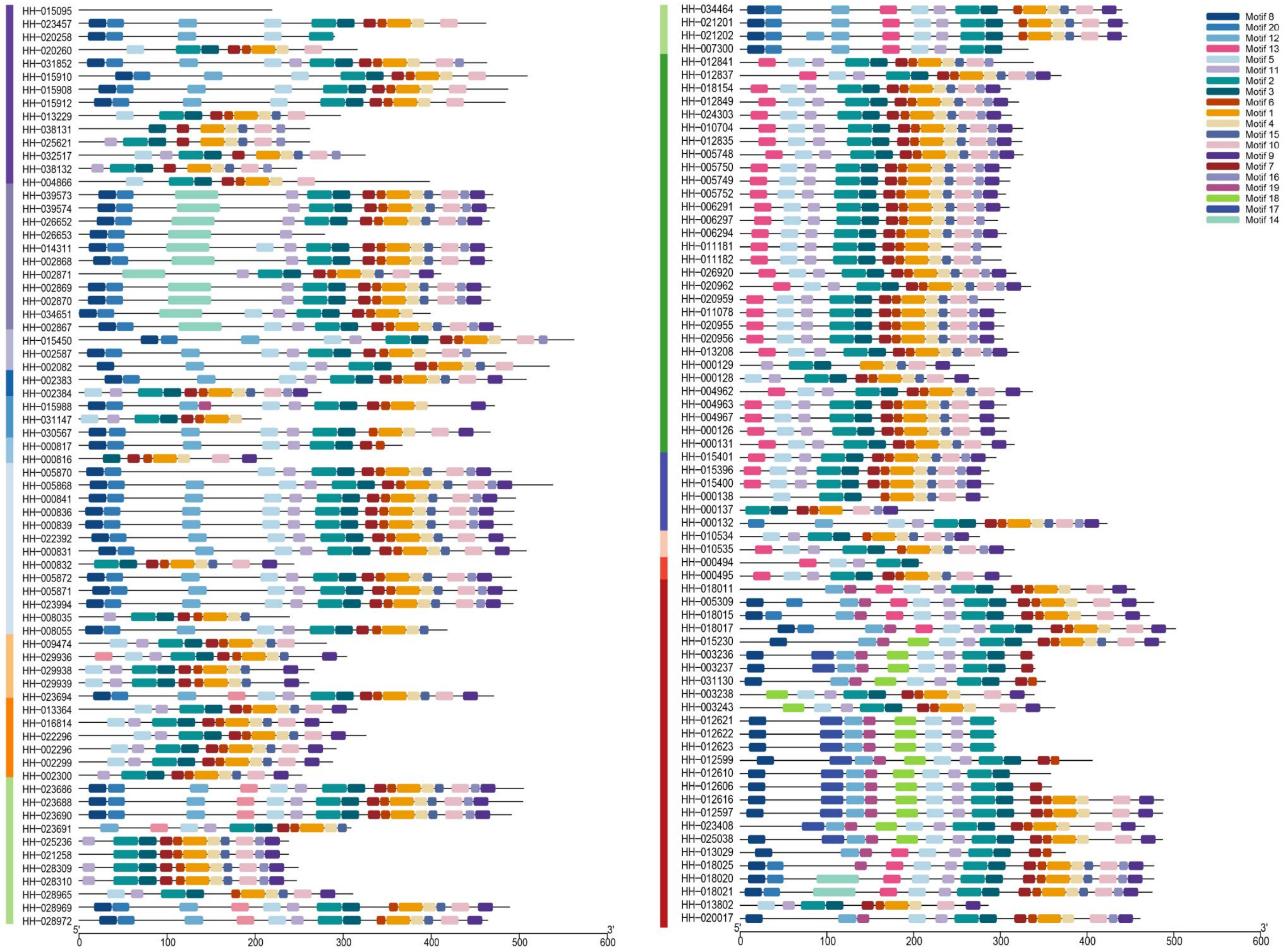

Supplementary figure S5

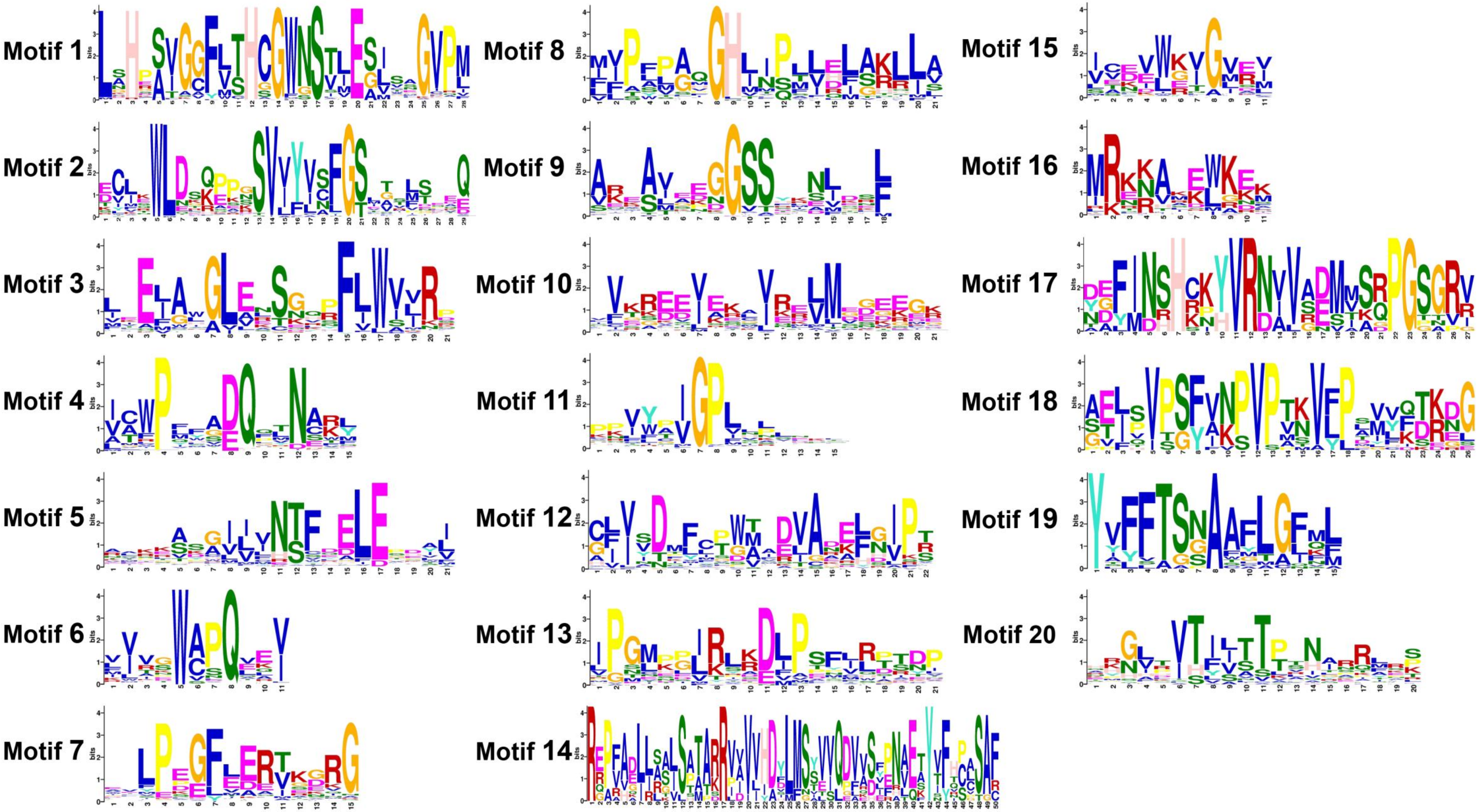

Supplementary figure S6

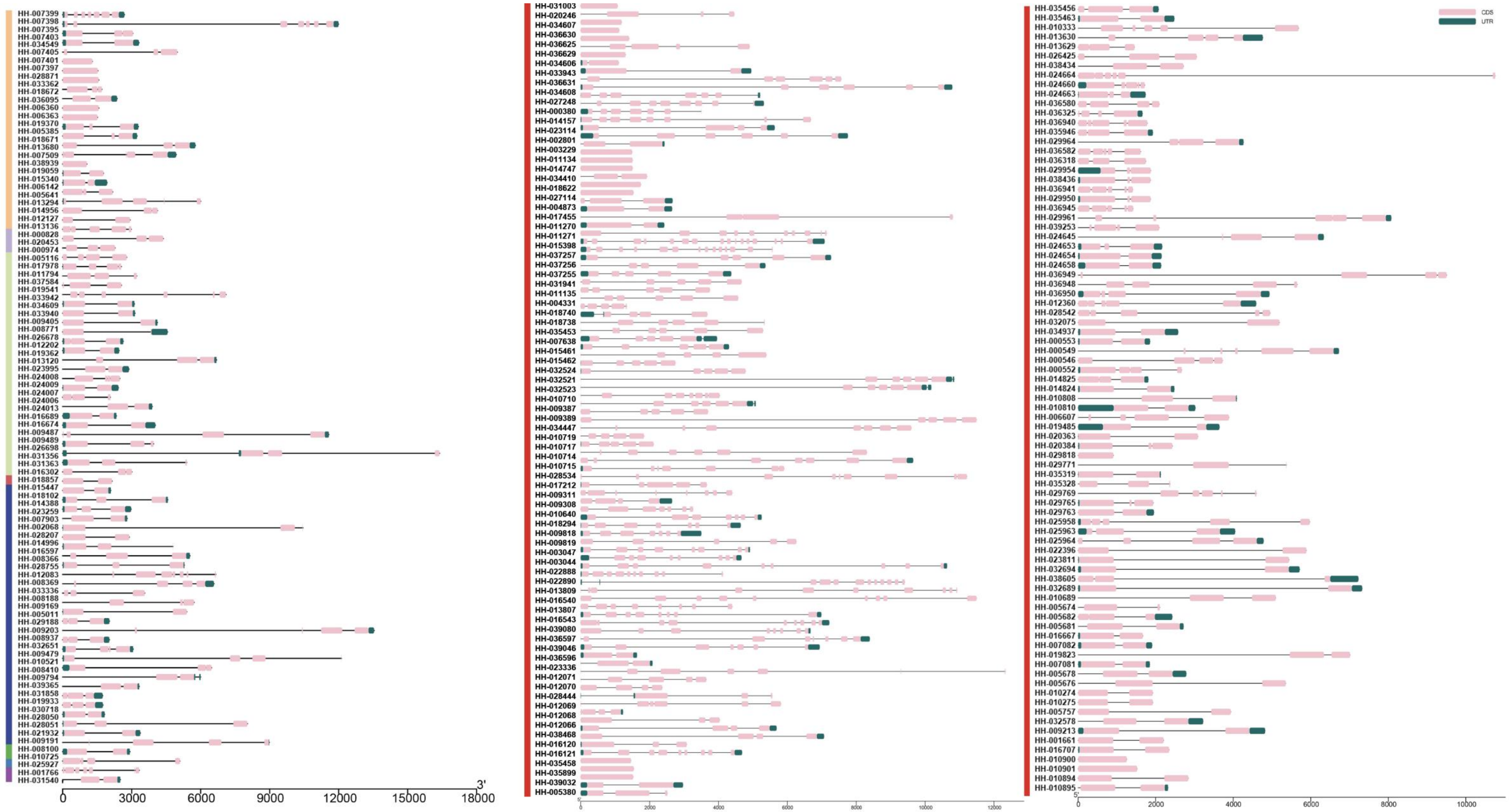

Supplementary figure S7

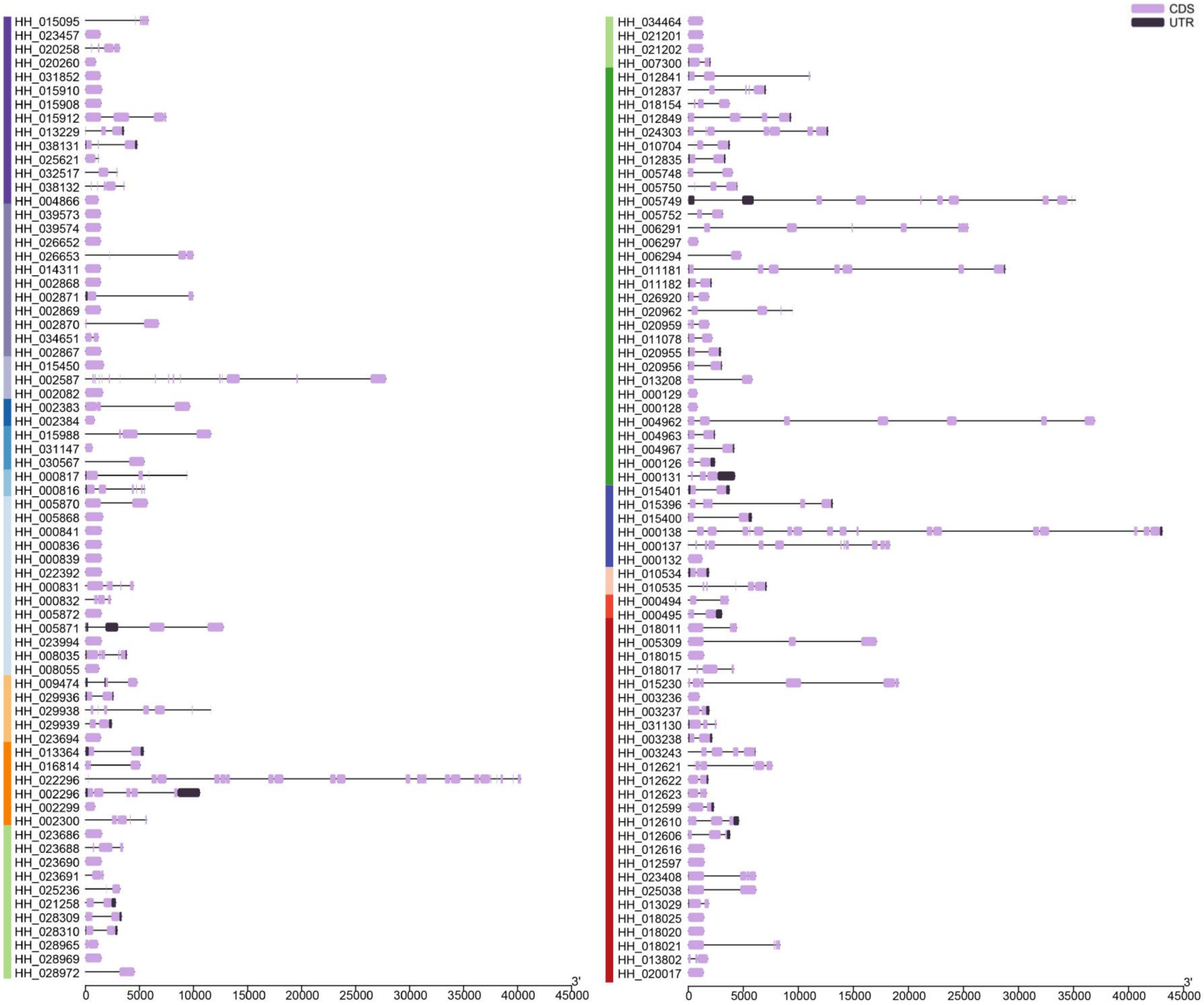

Supplementary figure S8

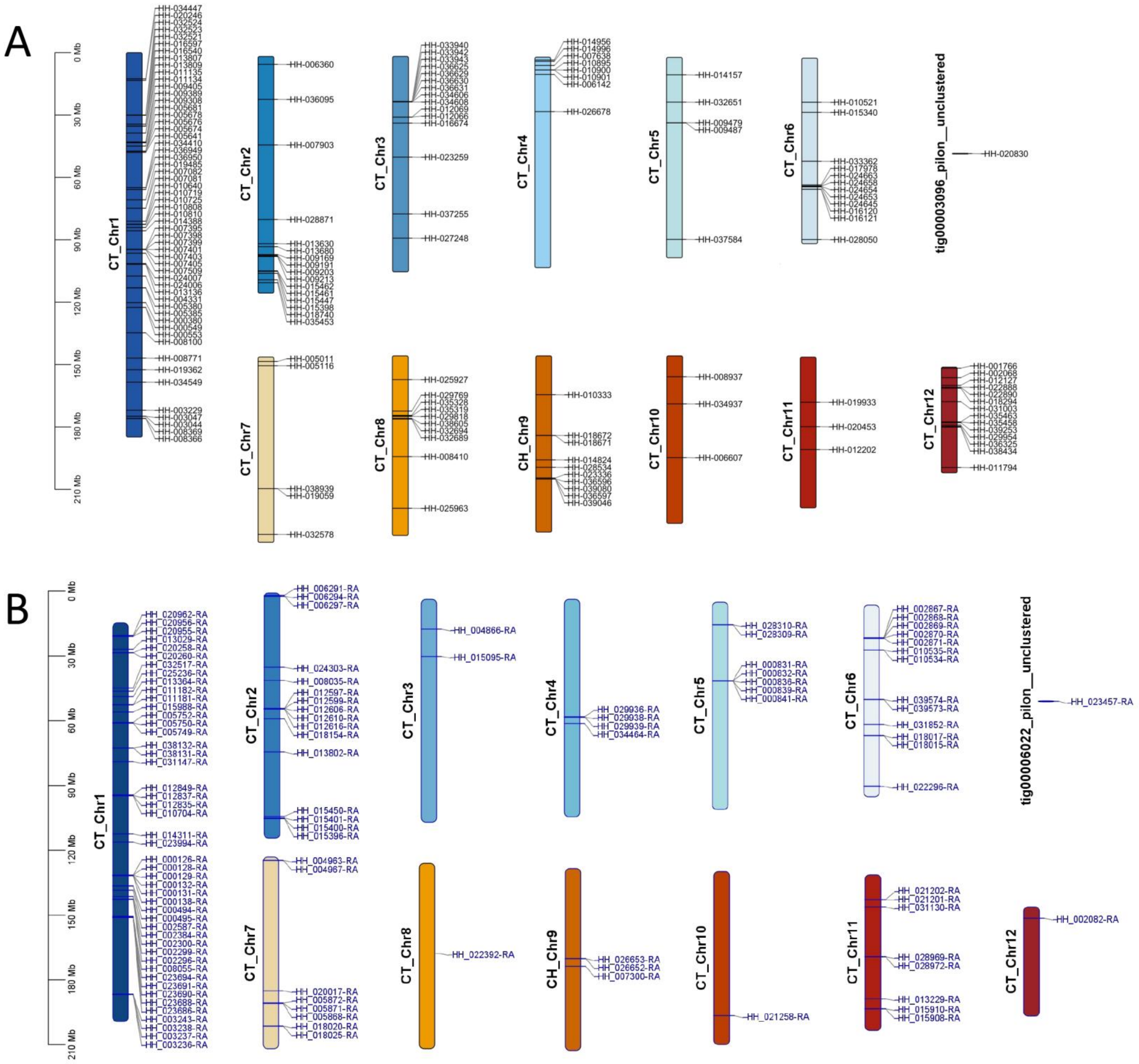

Supplementary figure S9

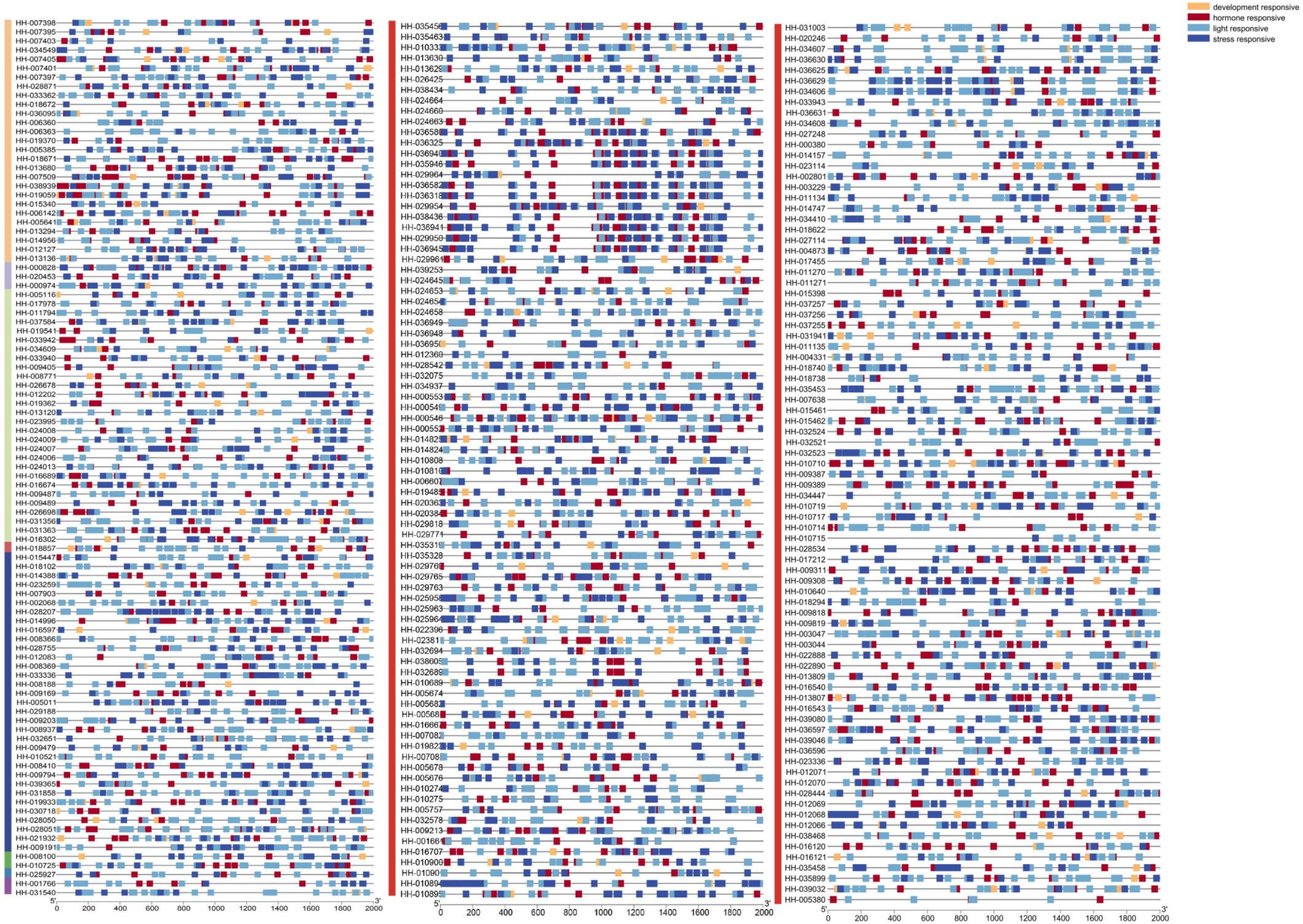

Supplementary figure S10

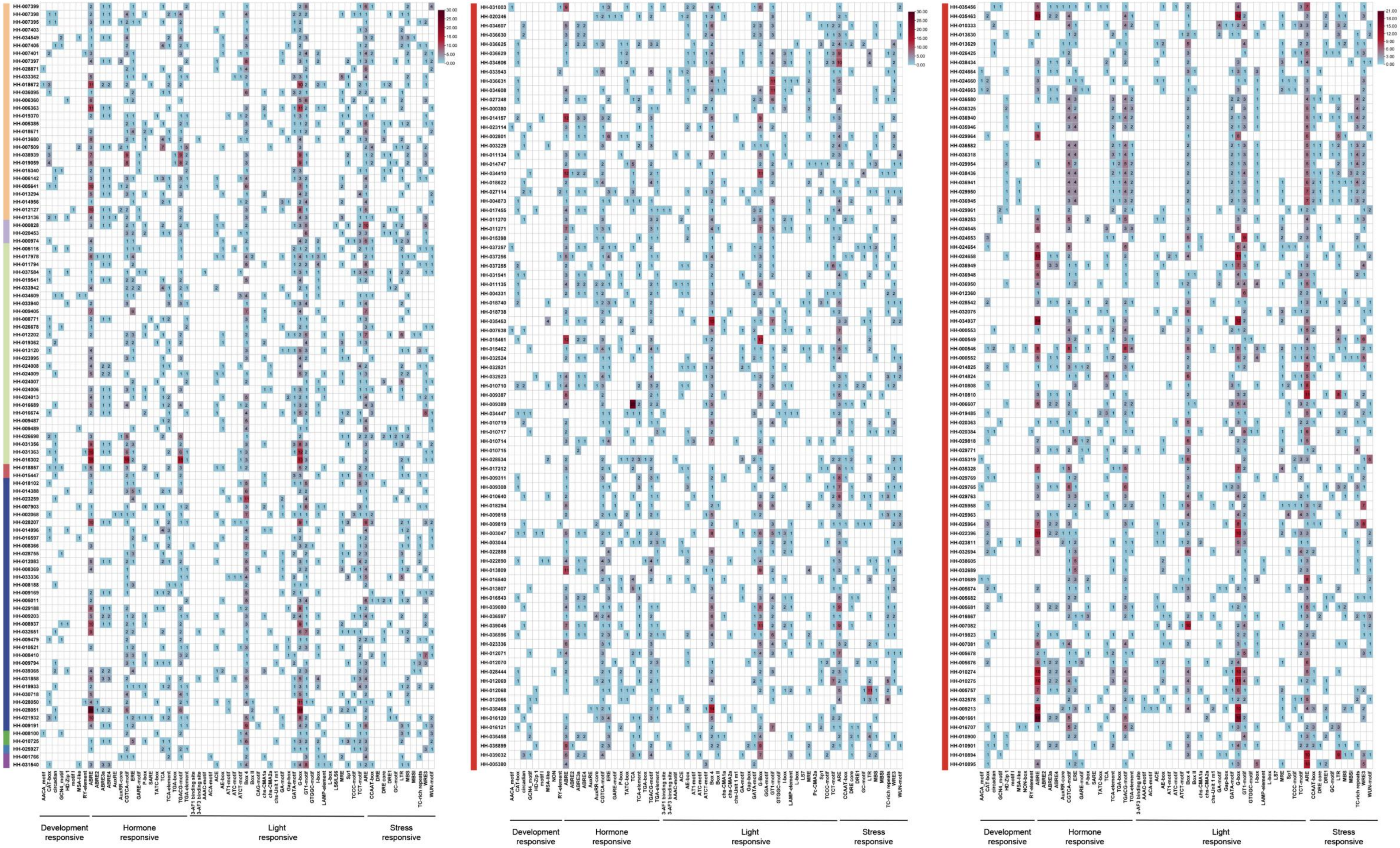

Supplementary figure S11

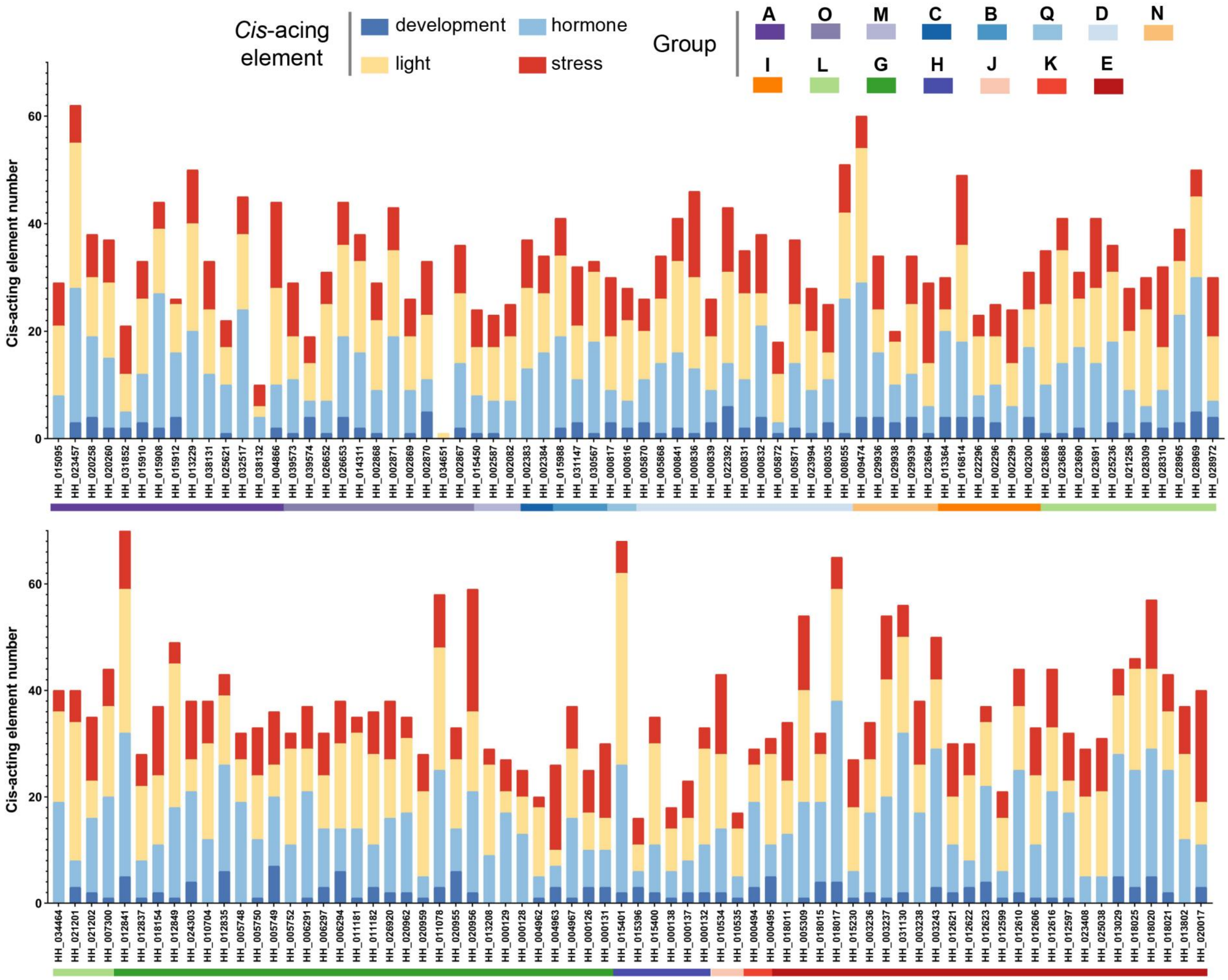

Supplementary figure S12

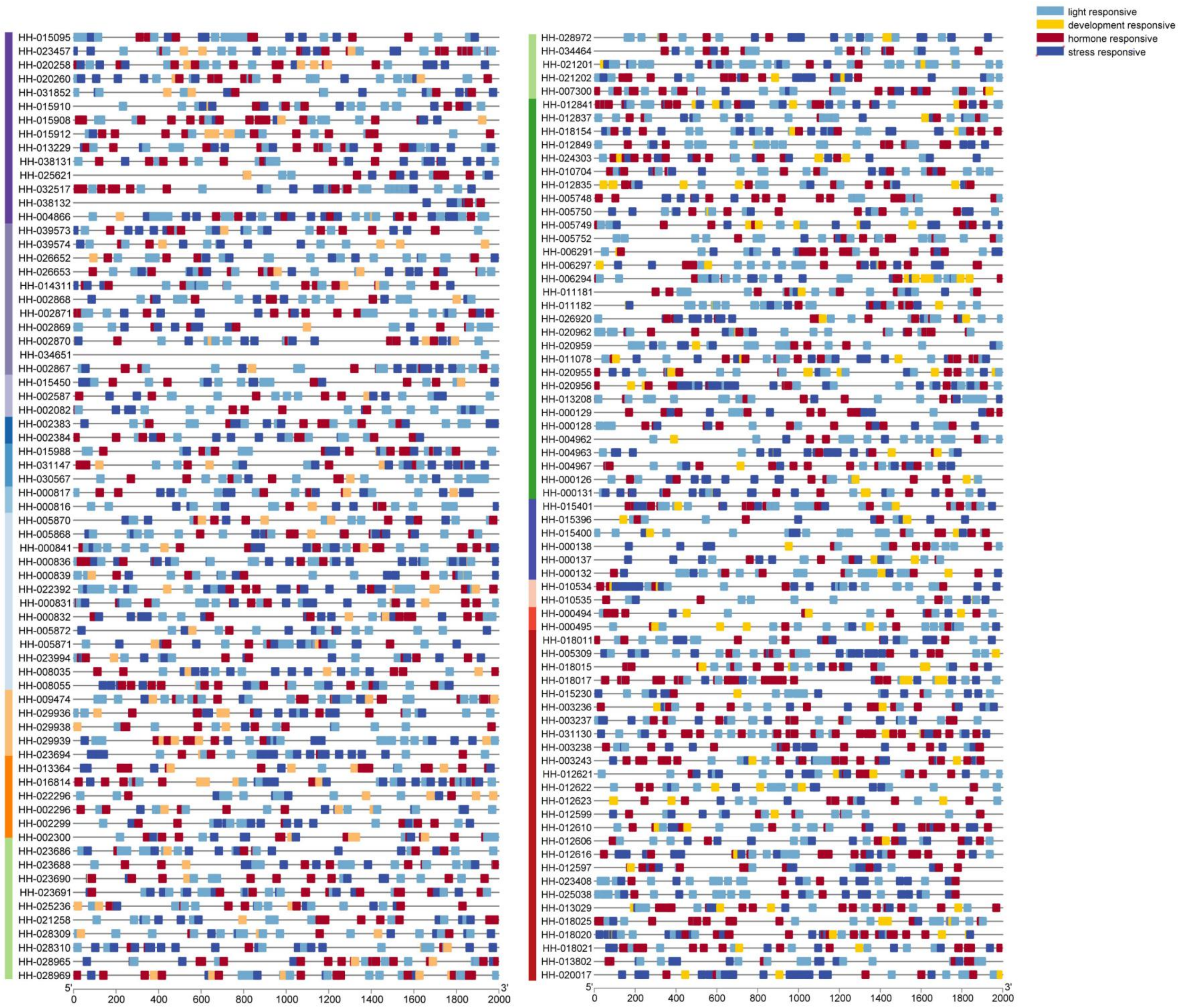

Supplementary figure S13

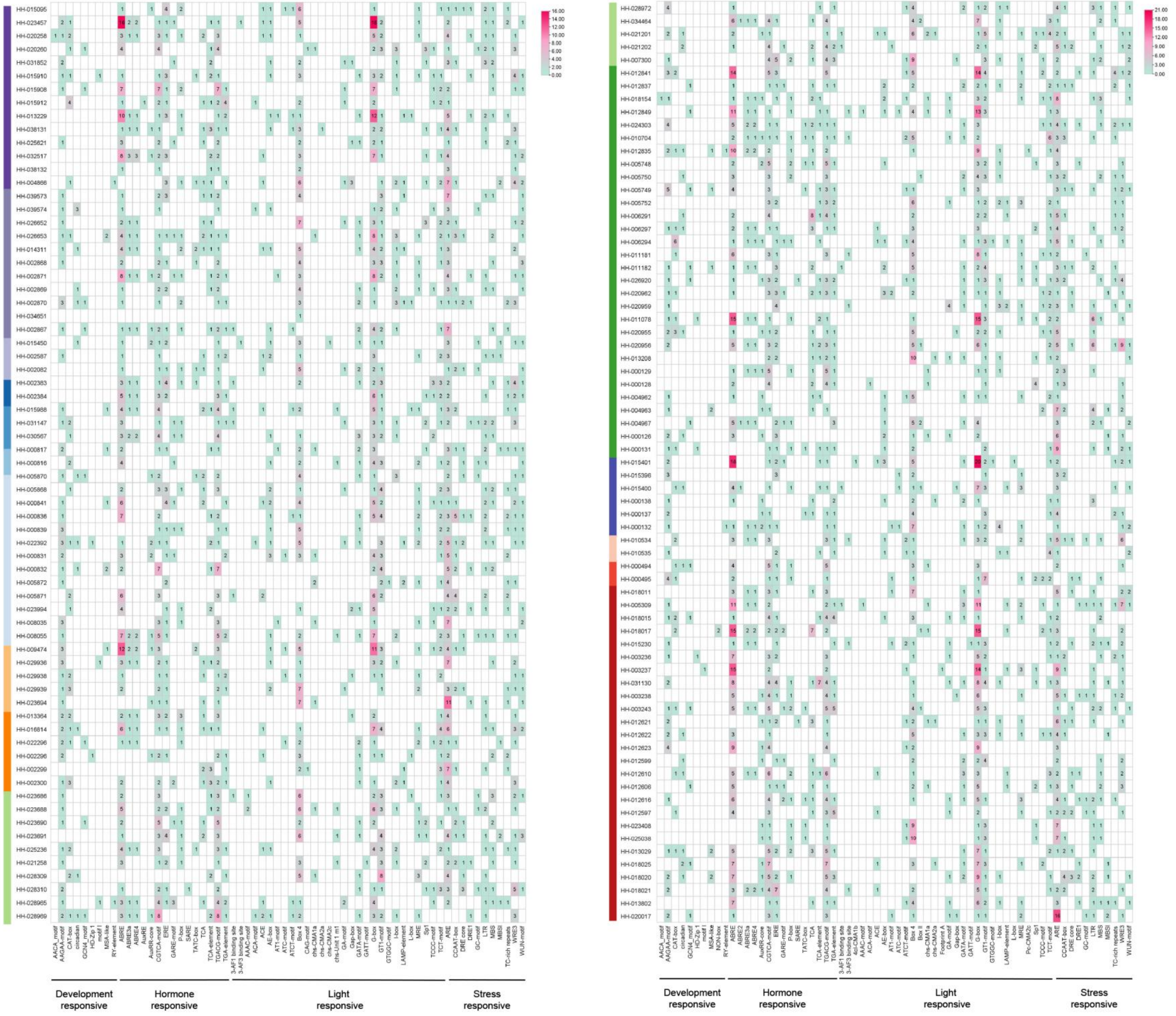

Supplementary figure S14

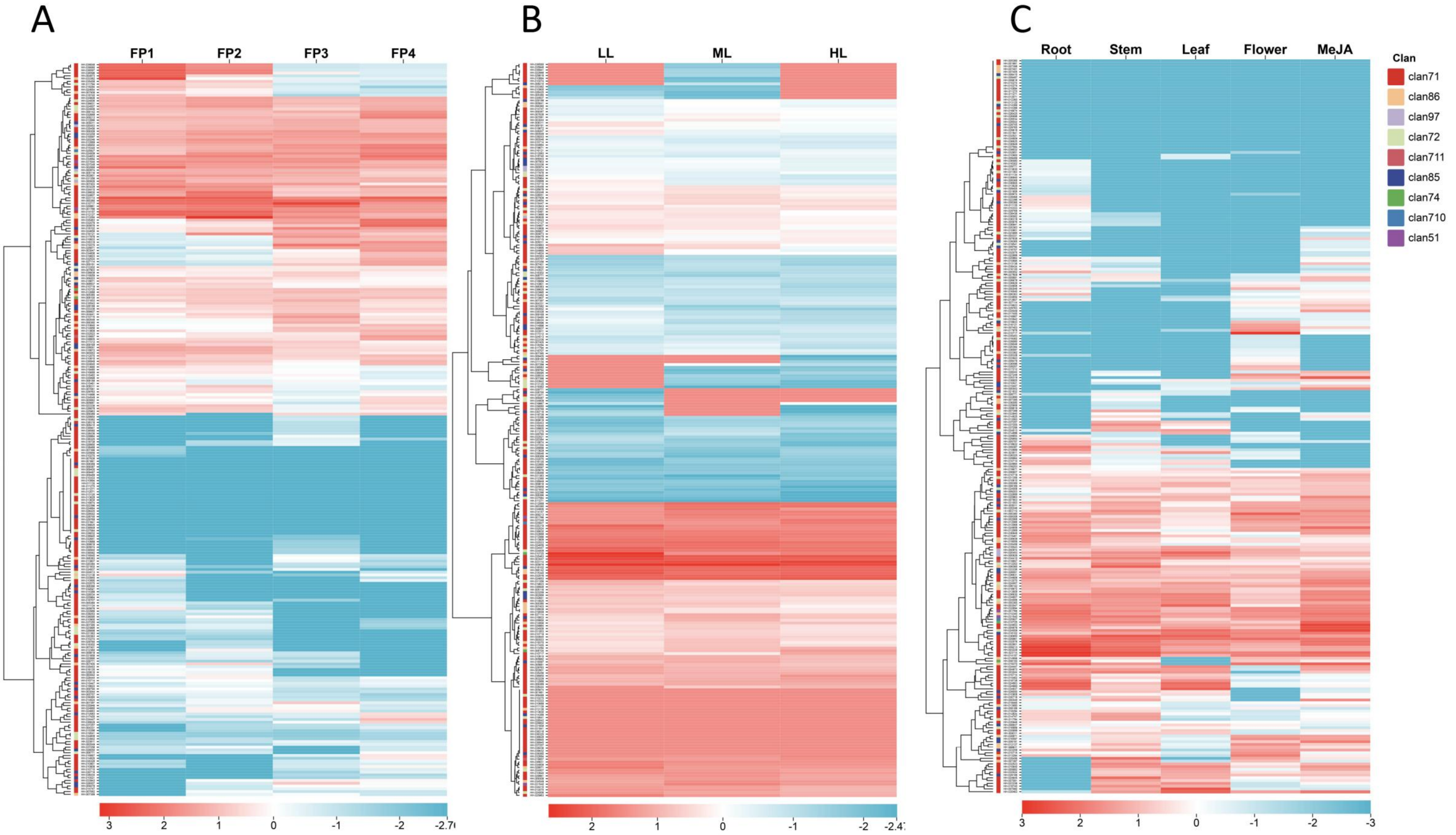

Supplementary figure S15

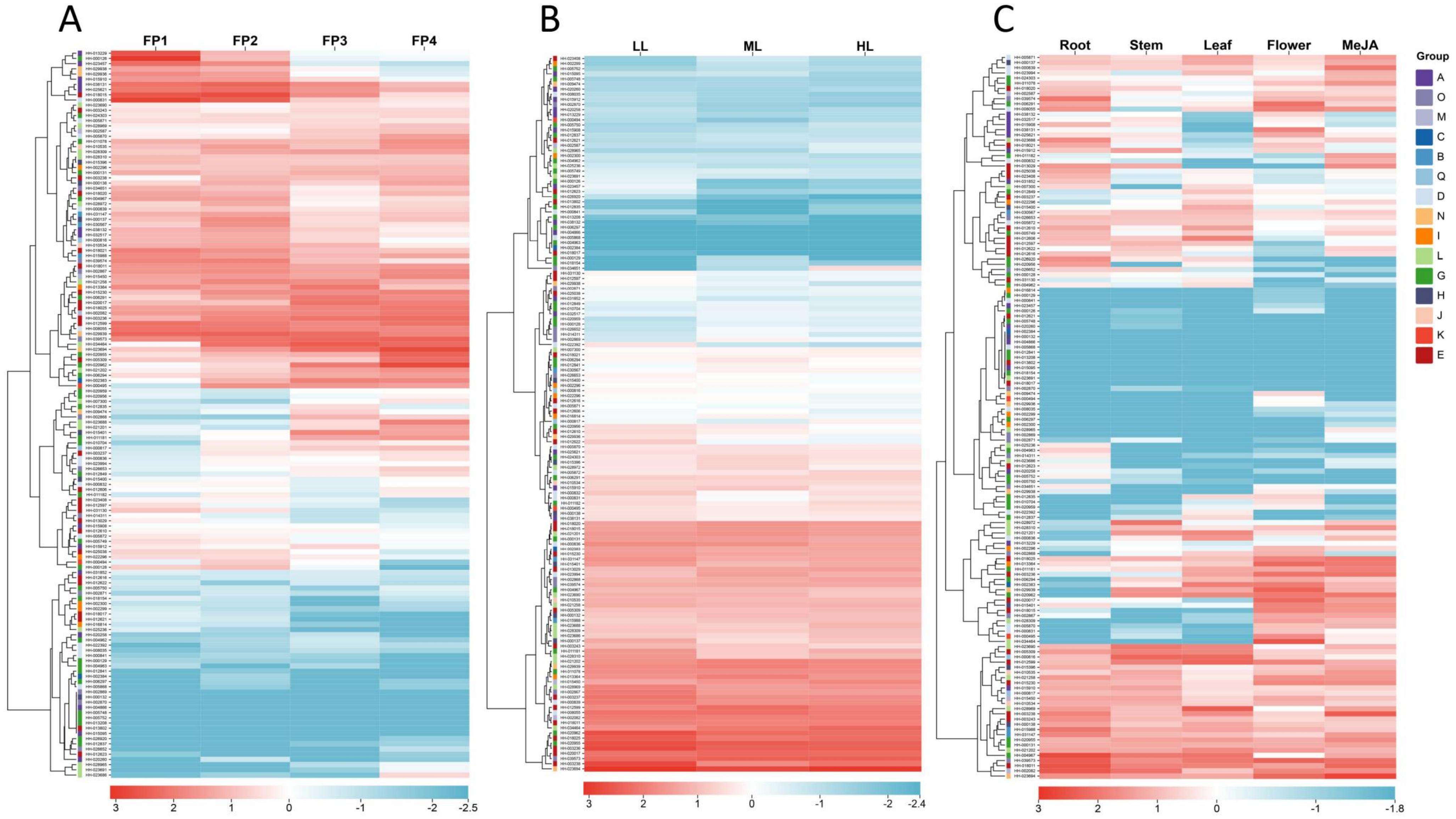

Supplementary figure S16

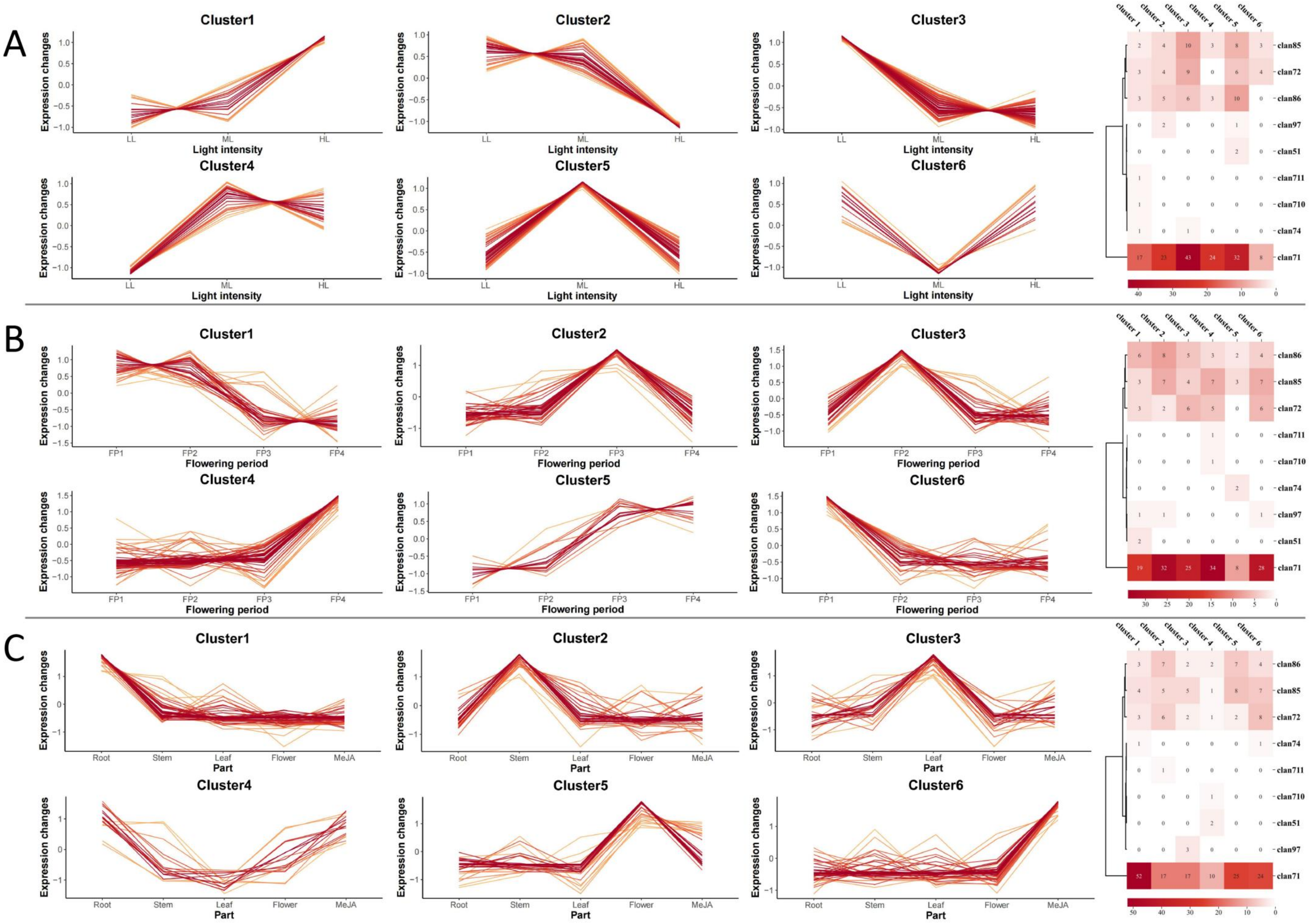

Supplementary figure S17

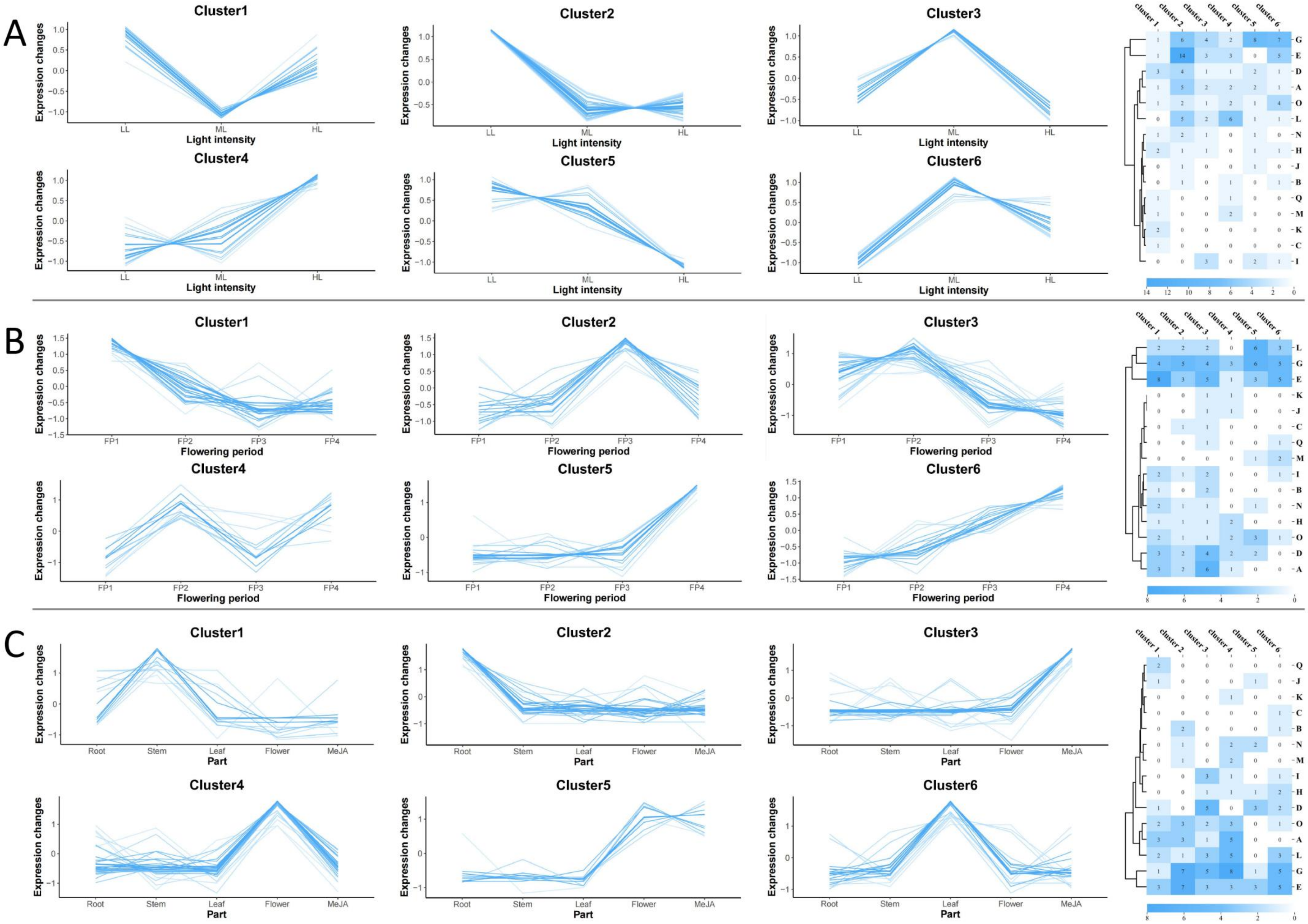

Supplementary figure S18

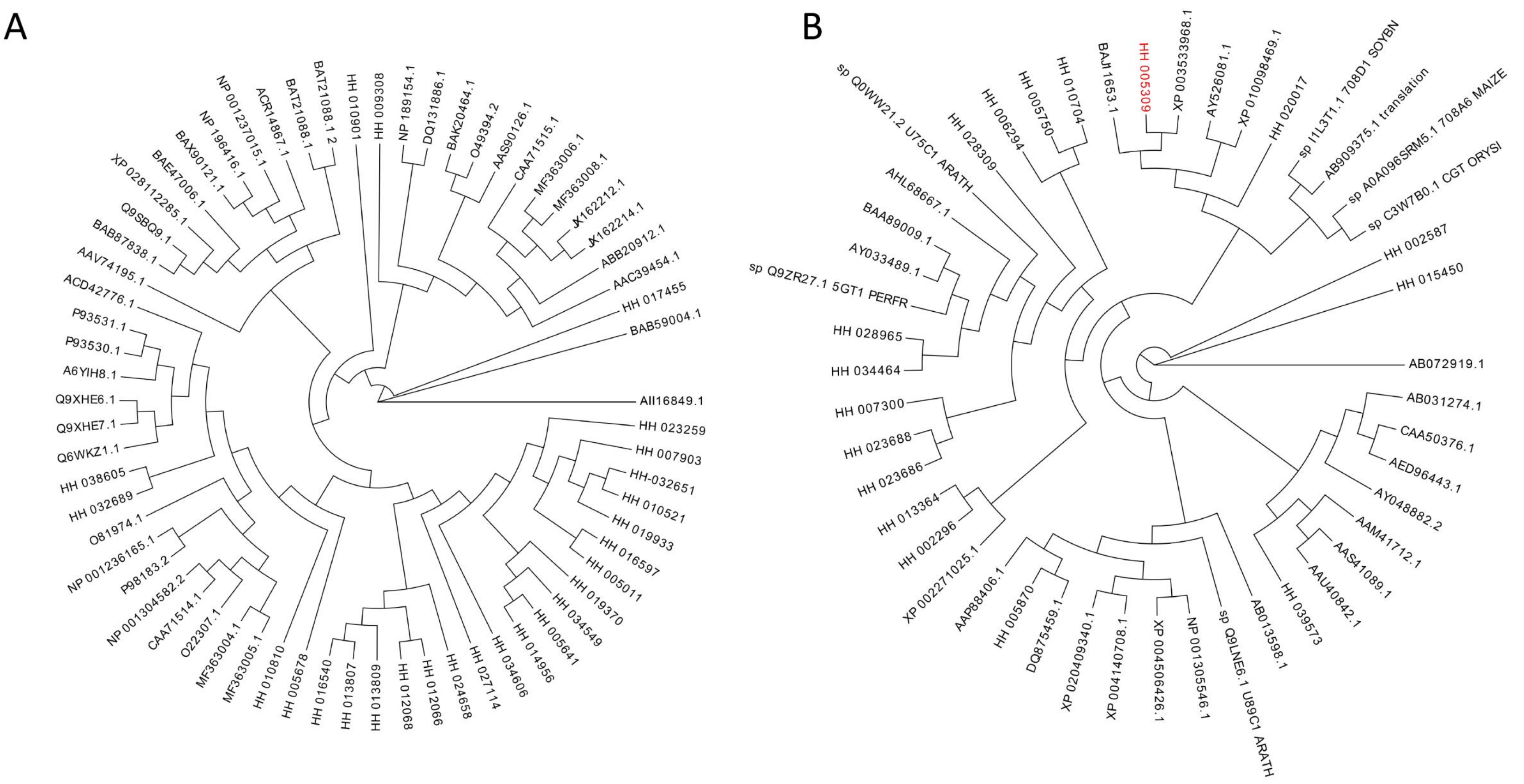

Supplementary figure S19

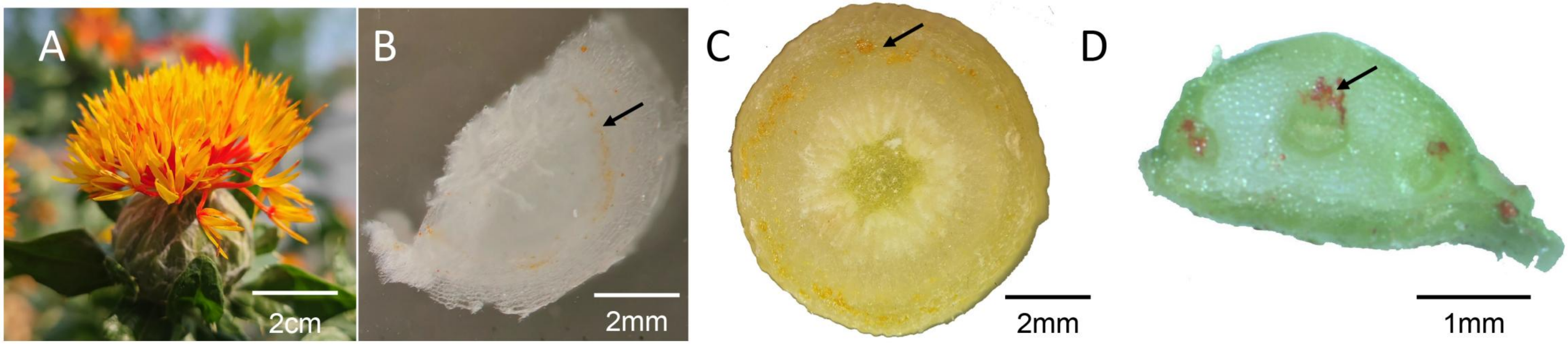

Supplementary figure S20

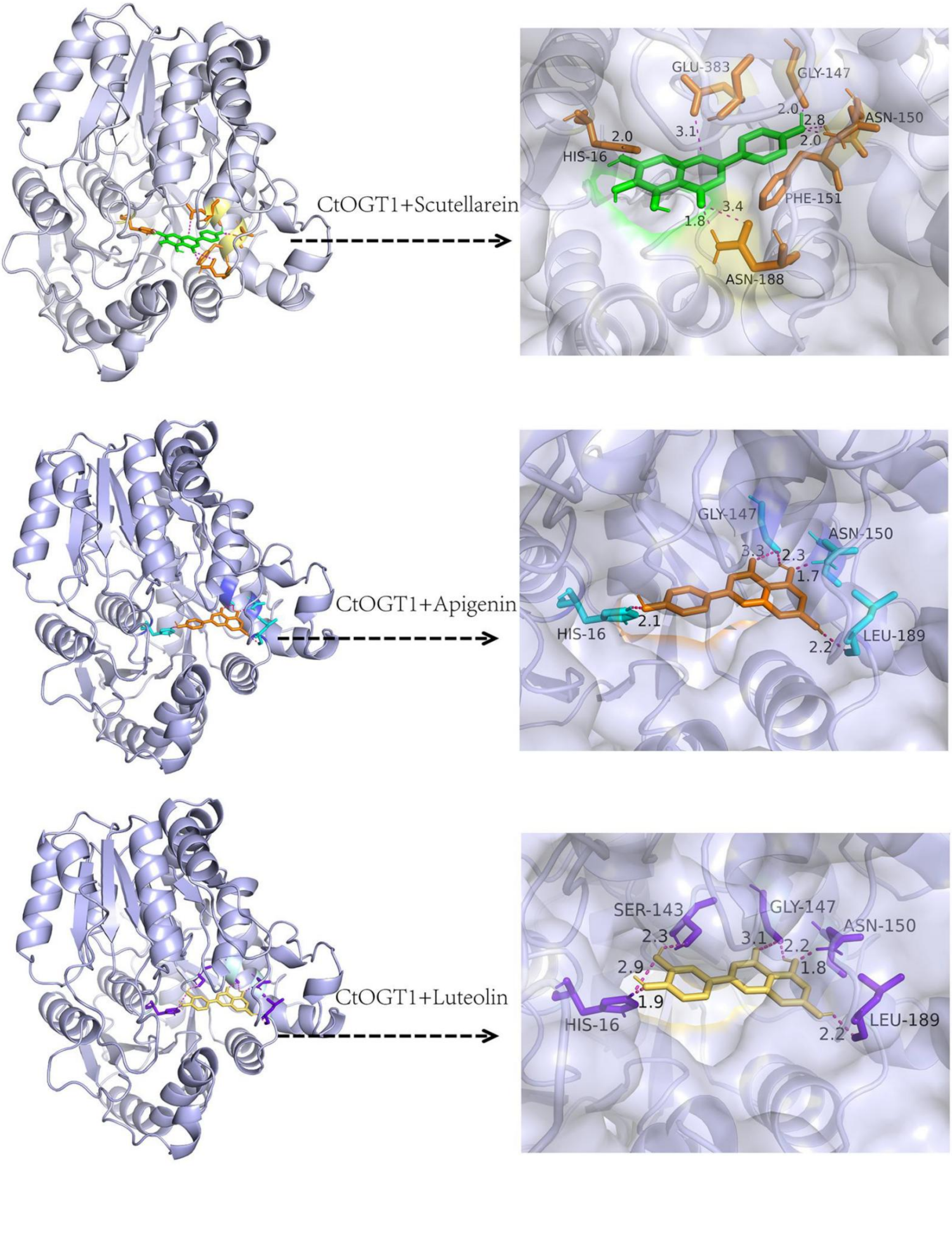

Supplement: Web_Material_uhae261 [file web_material_uhae261.zip › Supplementary Figures.pdf]
